# CRISPR-Cas12a fluorescence assays identify weedy *Amaranthus* species

**DOI:** 10.1038/s41598-026-49454-7

**Published:** 2026-04-28

**Authors:** Leonardo Galindo-González, Andrée Ann Dupras

**Affiliations:** Molecular Identification Research Lab, Ottawa Plant Laboratory, Canadian Food Inspection Agency, 3851 Fallowfield Road, Ottawa, ON K2J 4S1 Canada

**Keywords:** CRISPR-Cas12, *Amaranthus palmeri*, *Amaranthus tuberculatus*, Weed seeds, Species identification, Biological techniques, Biotechnology, Molecular biology, Plant sciences

## Abstract

Tall Waterhemp (*Amaranthus tuberculatus*) and Palmer Amaranth (*Amaranthus palmeri*) populations have developed multiple-herbicide resistance and impact the production of important crops, including corn, soybean and cotton. Morphological plasticity of these species, their persistence in the soil seed bank and their presence as contaminants during trade require efficient and sensitive methodologies to support their identification. Cas12 enzymes can be directed to a specific genomic region by a crRNA, cutting the DNA double strand and exhibiting collateral enzymatic activity on free-floating single-stranded DNA. This characteristic can be used to generate single-stranded oligonucleotide reporters bearing a fluorophore and a quencher, which will produce fluorescence when cut by Cas12. We designed CRISPR-Cas12 fluorescent assays to differentiate *A. tuberculatus*,* A. palmeri* and *A. palmeri*`s sister species (*A. watsonii*) from other *Amaranthus* species. Our assays for identifying the *A. palmeri + A. watsonii* clade and *A. tuberculatus* were 100% accurate when presented with blind samples of 14 *Amaranthus* species. Fluorescence could be detected using a blue light filter on a transilluminator within minutes. A preliminary recombinase amplification step to increase the limit of detection, and the Cas12 reaction, can be performed at room temperature and within an hour once DNA has been isolated.

**Supplementary Information:**

The online version contains supplementary material available at 10.1038/s41598-026-49454-7.

## Introduction

Morphological identification of plants is key in taxonomy and biodiversity research, as well as in the regulatory context. In this latter case, noxious species need to be rapidly identified and distinguished from non-regulated close relatives. However, morphological identification requires a high level of taxonomical expertise, and even then, it might not be sufficient when full specimens of a plant are not available, or when different developmental stages of a plant do not provide enough morphological characters to achieve species resolution.

The *Amaranthus* genus, with over 70 species, contains many weedy and invasive species, some of which have devastating effects on crops in North America^1–5^, reducing yields over 90% in maize^6^ and in the range of 30 to 80% in soybean^7^. Additionally, many of these species present resistance to multiple herbicide modes of action^2,5,8,9^, mounting pressure on management strategies.

Species like *A. palmeri* and *A. tuberculatus* have been regulated in North America and are of particular concern with major trading partners. Contaminated grain that arrived in Japan from the United States resulted in the quick establishment of palmer amaranth-resistant populations^10^, and an import ban imposed by China on Canadian canola after reporting palmer amaranth as one of the contaminants, resulted in losses of over 2 billion dollars to the Canadian economy^11^. Beyond contamination of trade seed, the incidence of climate change and the strong phenotypic plasticity of these weedy amaranths^1,12^, result in a northward range expansion of these species, which will make detection and eradication even more difficult.

The presence of *Amaranthus* weed seeds in the soil seed bank can be assessed by collecting soil cores and growing the seeds for weeks or months to have a sense of the prevalence of these species. In the meantime, detection during trade is performed by seed analysts who try to detect a target weed species among thousands of other seeds. Therefore, more efficient methodologies need to be developed to increase the efficiency of identification of these noxious species.

An alternative to support morphological characterization is the development of molecular tests that are based on DNA fingerprints (DNA barcodes) that belong exclusively to each species. Standard DNA barcoding with regions that oscillate between 300 and 800 bp is a common practice for plant species identification. Once the genomic information of the DNA barcode identifying the species has been obtained, amplifying and Sanger sequencing the region can be used for species identification when unknown samples are received. This process can be done in one to two days in laboratories with standard molecular biology equipment, including a genetic analyzer for sequencing. However, the process of species identification can be further accelerated by taking the available genomic information to design a rapid diagnostic test.

CRISPR-Cas systems have revolutionized the way we do molecular biology. CRISPR-Cas has been used for mutating genes, validating their predicted functions, regulating expression levels, and correcting detrimental mutations causing disease^13^. In plants, these enzymatic engineered systems have been used to increase plant productivity, protection against biotic and abiotic stress, and prospects now exist to increase photosynthetic capacity, eliminating pests through gene drives and molecular detection of variants^13^.

New members of the Cas family are often discovered, bearing novel functions or combining known functions with new ones. One of such enzymes is Cas12a (previously known as Cpf1), which some years ago was shown to have indiscriminate collateral cleavage (trans) activity on single-stranded DNA (ssDNA) after binding and cleaving DNA target sites that are complementary to a CRISPR RNA (crRNA)^14^. The sensitivity and accuracy of this system were tested through the development of HOLMES (a one-HOur Low-cost Multipurpose highly Efficient System)^15^, a method where a ssDNA bound to a fluorescent probe and a quencher, produced fluorescence when collateral cleavage took place. A second methodology using Cas12a, called DETECTR (DNA Endonuclease-TargetEd CRISPR Trans Reporter), used the same premise, pairing the enzyme’s collateral activity with isothermal amplification to reach attomolar-scale detection of human papillomavirus^16^. HOLMES and DETECTR followed a previous similar implementation (SHERLOCK—Specific High-Sensitivity Enzymatic Reporter UnLOCKing) that uses Cas13a to target viral RNA (instead of DNA), but bears similar collateral activity^17^.

HOLMES and DETECTR, and variations of these methodologies, provide single-base resolution and can be used for molecular diagnostics. For example, traditional PCR and recombinase amplifications were combined with a CRISPR-Cas12a to detect as few as 200 copies of white spot syndrome virus targets from shrimp^18^. Reverse transcription-loop-mediated isothermal amplification (RT-LAMP) was combined with a CRISPR-Cas12 assay to detect SARS-CoV-2 both by a fluorescence assay and using a lateral flow strip^19,20^. In food safety, this system has been tested to monitor genetically modified organisms, food-borne pathogens and animal-derived ingredient detection in search of adulteration^21^. In the field of plants, Cas12 fluorescent reporter tests were generated for genetically modified soybean and rice^22,23^, and also to detect the rice pathogen *Magnaporthe oryzae*^23^, and *Phytophthora ramorum*, a forest pathogen known as the causal agent of sudden oak death^24^. Both fluorescent and strip assays have been designed to increase efficiency and sensitivity in detecting plant viruses. Lately, a Cas12 method was implemented both with fluorescence and as a lateral flow assay to detect the seed-borne parasitic nematode *Aphelenchoides besseyi*, which is the causal agent of rice white tip disease^25^.

Despite the multiple applications offered by this technology, CRISPR-Cas12-based detection has only been used once before in the identification of closely related plant species^26^. In the regulatory context, monitoring of imported commodities is crucial to prevent the introduction of noxious and invasive species into the country. Likewise, methodologies to rapidly and efficiently detect exports containing plant species that are regulated in other countries are essential to comply with trade regulations. Molecular diagnostics methodologies provide an efficient and sensitive avenue to support the detection of species of concern and distinguish them from closely related species, even when a full-grown plant specimen may not be available.

Here we used LbCas12a from *Lachnospiraceae bacterium*, in combination with a preliminary recombinase amplification step to distinguish plants from the *Amaranthus* genus, which contains species of regulatory concern (*A. palmeri* and *A*,* tuberculatus*). The combination of Recombinase Polymerase Amplification (RPA) with a CRISPR-LbCas12a reaction using a 5-nucleotide AT-rich fluorescent reporter results in sample detection under an hour, with reactions that can take place at room temperature and without the aid of any additional equipment.

## Materials and methods

### Plant material

*Amaranthus* spp. plant material for standardizing our CRISPR-Cas12 assays comprised 64 samples and 17 species obtained from collaborators at Agriculture and Agri-Food Canada, Harrow, ON, and from the Germplasm Resource Information Centre (GRIN-Global), which is part of the U.S. National Plant Germplasm System. Sixty-three samples were obtained as seed, and a single sample was from the Genotyping and Botany private herbarium at the Canadian Food Inspection Agency (CFIA) (Supplementary Table 1).

Blind *Amaranthus* spp. samples were provided by Dr. Ruojing Wang (Seed Science and Technology Section at the CFIA, Saskatoon, SK), Dr. Sandra Flores-Mejia (CEROM, Saint‑Mathieu‑de‑Beloeil, QC) and Dr. Marie-Claude Gagnon (Genotyping and Botany Lab at the CFIA, Ottawa, ON). These blind samples corresponded to 63 tubes with 63 individual seeds (Saskatoon), 20 tubes with 20 individual seeds (Saint‑Mathieu‑de‑Beloeil) and 35 tubes with individual seeds/other plant tissue (Ottawa).

Seeds provided by AAFC (Harrow—Supplementary Table 1) were morphologically identified by a former Weed Ecology Scientist at AAFC Harrow (Dr. Susan Weaver). Seeds from *A. powellii*,* A. retroflexus*,* A. albus*,* A. blitoides* and *A. hybridus* were collected in Harrow, *A. rudis* from Cottam/Essex County, and *A. spinosus* was collected in an unspecified location from southern Ontario. Material coming from the U.S. National Plant Germplasm System was identified by David Brenner (Plant Germplasm Curator) and Donald Pratt (support Graduate student from Iowa State University) and can be queried through the GRIN portal (https://npgsweb.ars-grin.gov/gringlobal/search) using the IDs from Supplementary Table 1. All accessions can be ordered as seed for research purposes, and were obtained through CFIA import permits. Accessions AMES 5152, AMES 5303, PI658730, PI690570, PI632236, PI633586, PI633587, PI633593, AMES 5328, AMES 5150 have corresponding herbarium vouchers from the National Arboretum, in Washington D.C. Blind samples from the Seed Science and Technology Section at the CFIA, were identified by Jennifer Neudorf (CFIA Technologist), and belong to the private Canadian National Seed Herbarium. Blind sample seeds provided by CEROM were collected across Quebec with the exception of samples 71 and 73 (see Table 2 below), which were provided to CEROM by Professor Muthukumar V Bagavathiannan (Texas A&M), and sample 75 which was provided by Dr. Peter Sikkema (University of Guelph). Original identification of CEROM samples was performed by staff at CEROM and using a molecular test provided by the Laboratoire d’Expertise et de Diagnostic en Phytoprotection (LEDP). Blind samples provided by CFIA’s Genotyping and Botany lab were identified by Andreanne Charron, Adèle Julien and Alexandre Blain, who worked as botanists at different times for the lab. Samples 84, 85, 86, 87, 94, 95, 99, 101, 103, 105 and 107 (Table 2, below) have corresponding vouchers from the private CFIA GenoBot Herbarium.

### DNA extraction and quantification

For assay development, five to 10 seeds of each one of the accessions tested were homogenized in tissue disruption tubes (containing ballcone-shaped beads) using the TissueLyzer II, run at 24 Hz for 2 min, followed by a centrifugation step at 12,000 x g for 2 min. DNA was then extracted using the DNeasy Plant Pro kit (Qiagen) with the manufacturer’s recommendations. Extracted DNA was quantified using the Quibit 1X dsDNA HS assay kit (Thermo Fisher Scientific). Blind samples were extracted using the same protocol above, but for individualized seeds.

### CRISPR-LbCas12a assay design

CRISPR RNAs (crRNAs) target regions were selected after aligning full chloroplast genomes of 28 *Amaranthus* samples corresponding to 15 different species. These full chloroplast assemblies were produced in our lab and will be shared in an upcoming publication discussing DNA barcoding in *Amaranthus* species. Additional non-redundant chloroplast genomes previously used for a comparative analysis of dioecious *Amaranthus*^27^, were added to the alignment to further validate the specificity of the crRNA target regions.

**Fig. 1:**
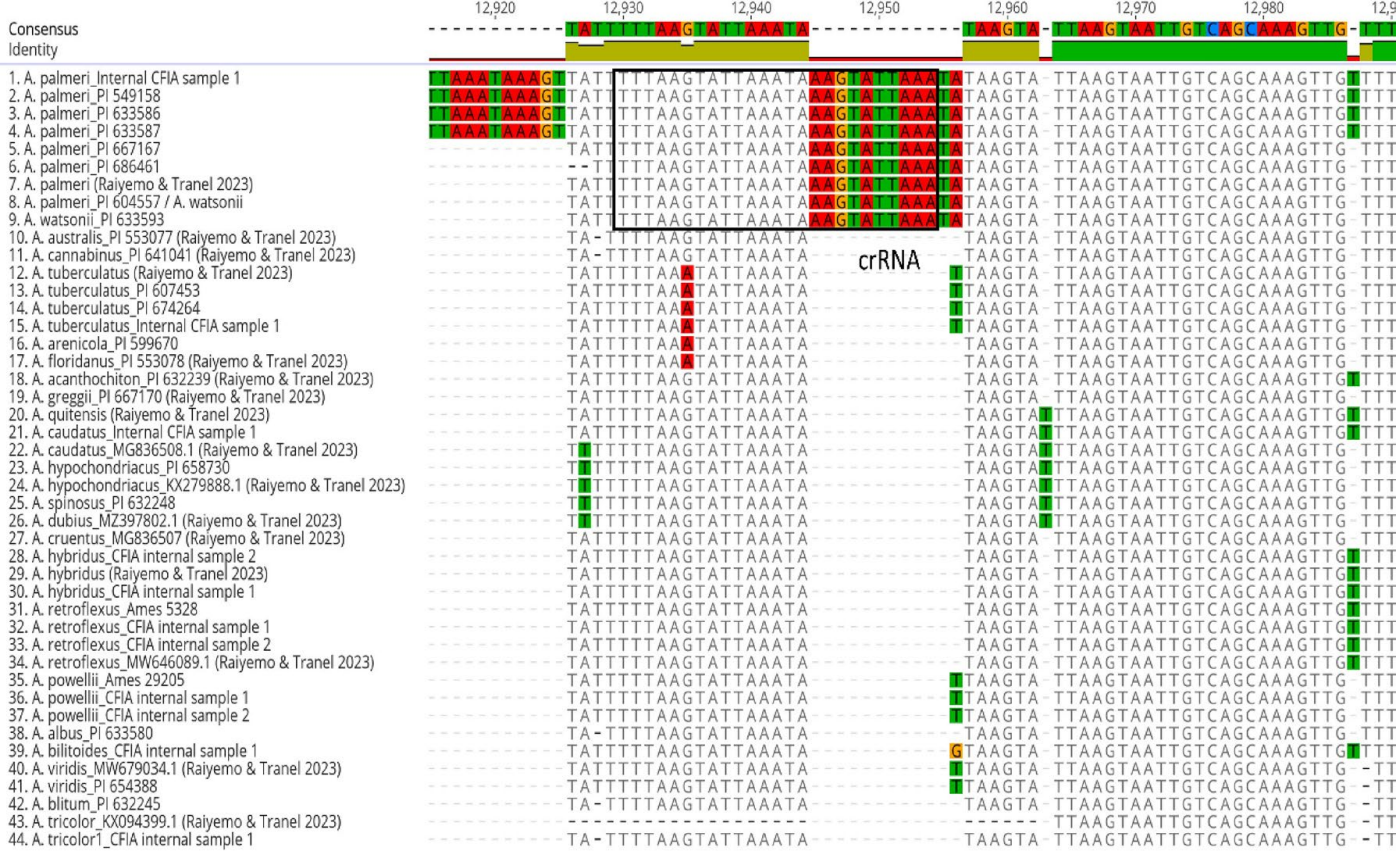
Alignment of a section in the chloroplast genome (*atpF* gene) selected to design an *A. palmeri + A*. *watsonii*-specific crRNA (boxed). The crRNA is in the complementary strand and starts with a PAM sequence needed for Cas 12 binding (TTTV). The alignment includes 28 chloroplast genomes previously assembled in our laboratory, plus genomes used in a previous study^27^. According to multiple molecular analyses from our lab and phenotyping of grown seeds from accession PI 604557 obtained from GRIN-USDA, this accession is likely *A. watsonii* or a hybrid population and not *A. palmeri.* This accession has since been retired from the GRIN germplasm system and re-classified as a mix or hybrid of these two sister species (https://npgsweb.ars-grin.gov/gringlobal/search).

Geneious Prime (v 2022.0.2) was used for aligning assembled chloroplast genomes using MAFFT with a 200 PAM matrix, k = 2, gap penalty of 1.53 and an offset value of 0.123. CRISPR-Cas12 (Cpf1) target sites were also predicted using Geneious, using TTTN as the protospacer adjacent motif (PAM) and a length of 21 nucleotides for the crRNA target region. Alignments were scanned visually to find species-specific polymorphic regions that overlapped the predicted crRNA target regions. A region corresponding to the *atpF* gene with polymorphisms that were specific only to *A. palmeri + A. watsonii* (Fig. 1), was bound to a stem loop to generate the first crRNA (Apal+Awat_crRNA: *UA AUU UCU ACU CUU GUA GAU* AUA CUU UAU UUA AUA CUU AAA)—the stem loop that is common to all crRNA is italicized.

To distinguish *A. palmeri* from *A. watsonii*, we aligned the accessions corresponding to these two species and found a suitable polymorphic region for crRNA design in the intergenic region between *trnS-*GAA and *rps4* (Apal_vs_Awat_crRNA: *UA AUU UCU ACU CUU GUA GAU* UUC GAU UUC GAA UUG CUG UGG) (Supplementary Fig. 1). To distinguish *A. tuberculatus* from other Amaranthus species, we used an intergenic region between *trnG-*GCC and *trnFm-*CAU (Supplementary Fig. 2). This crRNA was designated as Atub_crRNA-2 (*UA AUU UCU ACU CUU GUA GAU* UCU UAU AGU AAA UAA AAU UAC).

### Recombinase polymerase amplification (RPA)

For isothermal amplification of the target regions, we designed RPA primers for the three assays that would amplify all available *Amaranthus* species. RPA primers were designed based on recommendations outlined for the TwistAmp DNA Amplification Kit used (https://www.twistdx.co.uk/support/rpa-assay-design/). For the *A. palmeri* + *A. watsonni-*specific assay, we used RPA fw primer: CACTAATCAAGACTAGAATATTTGGAGGACTCTTC, and RPA rv primer: TAAATGCCAAAGGCTGAGTCGACGACCTAC. Recombinase primers to amplify the region to distinguish *A. palmeri* from *A.watsonii* were: RPA fw primer: TCTGAGTCATAATCAGTGTGTAGGAGAGAT, and RPA rv primer: TCCCATCGTGAACTTTCCTATTGATGTATCATAA. Finally, to amplify the region encompassing the binding site of the *A. tuberculatus-*specific crRNA, we designed RPA fw primer: GGTAAAATTTCTCTTTGCCAAGGAGAAGAC, and RPA rv primer: ACGGGTTCAAATCCTGTCTCCGCAAAATTT.

We used the TwistAmp Liquid Basic Kit (TwistDx) for recombinase-mediated amplification. RPA reactions were done in 50 µL following the manufacturer’s recommendations. Briefly, a pre-mastermix containing 25 µL of 2X reaction buffer, 0.9 µL of 10 mM dNTPs, 8.3 µL of water, 5 µL of 10x basic E-mix and 2.4 µL of each primer at 10 µM was vortexed and spun. Then 2.5 µL of the 20x core reaction mix was added to the tube lids, and then the tubes were closed and the solution mixed by inversion 10 times, followed by a brief spin. The 46.5 µL were then added to a 0.2 mL PCR tube, and 2.5 µL of 280 MgOAc and 1 µL of DNA (15 ng) were added to the lids but kept separate. After carefully closing the lids, the mixtures were spun and mixed six times by inversion. The tubes were spun down briefly, and the reactions were incubated at room temperature (~ 22 °C) for 20 min. For visualization by agarose gel electrophoresis, EDTA was added to each sample at a final concentration of 20mM and samples were incubated for 5 min before loading onto a 1.5% agarose gel.

### CRISPR-LbCas12a reaction

For the 50 µL LbCas12a-mediated reaction, 2 µL of the RPA amplification reaction (without purification) were mixed with 100 nM of LbCas12a (Integrated DNA Technologies), 100 nM of crRNA (Integrated DNA Technologies) and 1x NEB buffer 2.1 (New England Biolabs). The solution was spun for 10 s and incubated at room temperature for 25 min. Then we added 15 ng of the activator (amplified target DNA region), and 1 µM of the TA-rich oligo reporter (single-strand DNA or ssDNA)^28^ containing a FAM fluorophore and an IowaBlack quencher (/56-FAM/TT ATT /3IABkFQ/). Finally, 28 µL of water were added to the LbCas12a-crRNA ribonucleoprotein complex. The tubes with the mix of the enzymatic complex and targets were left at room temperature (~ 22 °C), and a picture was captured under a Gel Doc EZ Imager (Bio-Rad) every 5 min for an hour under a blue light filter (470 nm wavelength).

### Quantification of fluorescence

To test if visible qualitative changes represented a significant quantitative change in fluorescence, we tested our validated 64-sample set with the Apal+Awat_crRNA in triplicate, and quantified the level of fluorescence using a QuantStudio5 (Life Technologies—ThermoFisher Scientific). All conditions for recombinase amplification and CRISPR-Cas12 reaction were as described above, and fluorescent values were captured every 5 min for an hour. The QuantStudio qPCR was set to genotyping, and the cycling conditions included 60 cycles of 30 s at 37 °C, with fluorescence captured at each cycle, followed by 1 cycle of 2 min at 98 °C.

Triplicate technical replicates of raw fluorescent values were averaged and log10-transformed to be used on an R-script to test normality and homogeneity of variance, and then perform analysis of variance and post-hoc multiple comparisons (Supplementary Data 1).

### Blind sample testing

Blind samples were obtained from three different sources. Dr. Ruojing Wang from the CFIA Saskatoon Seed Science and Technology (SSST) lab provided 63 individual seed samples; Dr. Sandra Flores-Mejia (Centre de Recherche sur les Grains—CÉROM) provided 20 individual seeds; and Dr. Marie-Claude Gagnon (CFIA Genotyping and Botany Lab) provided 35 samples. DNA was extracted individually from each seed/tissue sample, and the DNA was used for all three CRISPR-Cas12 tests as described above.

### Limit of detection assay

To establish the limit of detection of our assay, we performed five DNA serial 1:10 dilutions, starting with 15 ng of DNA (standard amount of all experiments), in two accessions of *A. palmeri* and two accessions of *A. tuberculatus.* The target regions from the initial DNA sample (15 ng) and the six dilutions were recombinase-amplified and tested with our CRISPR-Cas12 species-specific assays as described above. A negative control of an *A. tuberculatus* or an *A. palmeri* sample was included for the *A. palmeri* + *A. watsonii* or the *A. tuberculatus*-specific assay, respectively.

## Results

### Assay validation

Full chloroplast genomes from 28 *Amaranthus* accessions corresponding to 15 species were previously assembled at our lab and used to identify potential polymorphic regions that could be targeted with a crRNA-Cas12 complex. Our assemblies were aligned with previously reported genomes^27^ to further support the specificity of the designed crRNA across a wider number of species and accessions within the genus. We selected a region within the chloroplast *atpF* gene showing polymorphism between the *A. palmeri* accessions (+ *A. watsonii*, the sister species of *A. palmeri*) and the rest of the species assembled (Fig. 1).

**Fig. 2:**
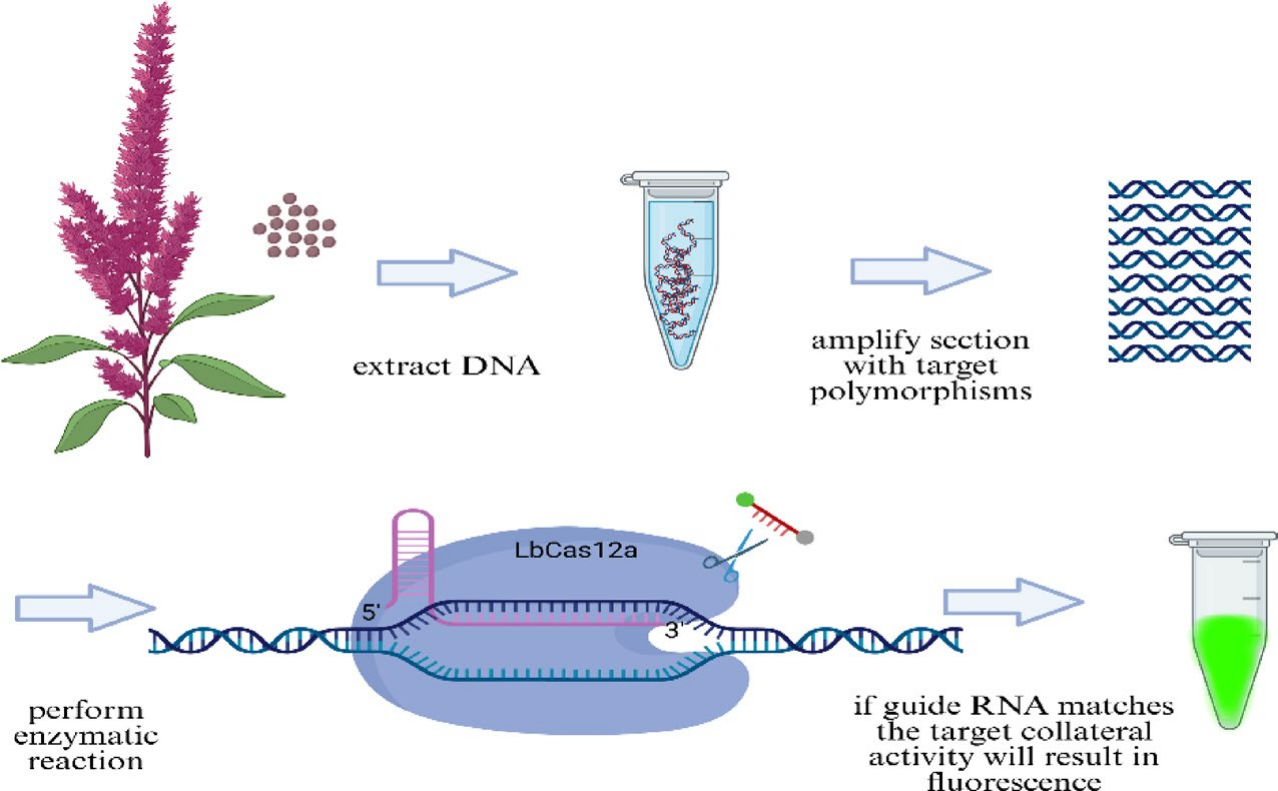
Diagram depicting the pipeline of sample processing to test for the presence of target sequences using Cas12 collateral activity. DNA is extracted from individual seeds or other tissues in *Amaranthus* plants, followed by amplification of the target region expected to bear the polymorphisms to distinguish different species. The amplified region is then mixed with the LbCas12 enzyme, the species-specific guide RNA (crRNA), and a small ssDNA probe with a fluorophore and quencher. If the crRNA-Cas12 complex finds the complementary DNA sequence, the collateral activity of the Cas12 enzyme will cut the ssDNA probe, releasing the fluorophore from the proximity of the quencher, resulting in a fluorescent signal. Created in BioRender. Galindo, L. (2026) https://BioRender.com/swg6xu3.

An RPA-mediated pre-amplification step on the target region containing the polymorphic region was performed for DNA extracted from 64 samples corresponding to 17 *Amaranthus* species, with multiple accessions tested (when available) for most of the species. The amplification products were mixed with LbCas12a, the crRNA, and a 5mer reporter containing a FAM fluorophore along with an IowaBlack quencher. A diagram of the experiment workflow is shown in Fig. 2.

**Fig. 3:**
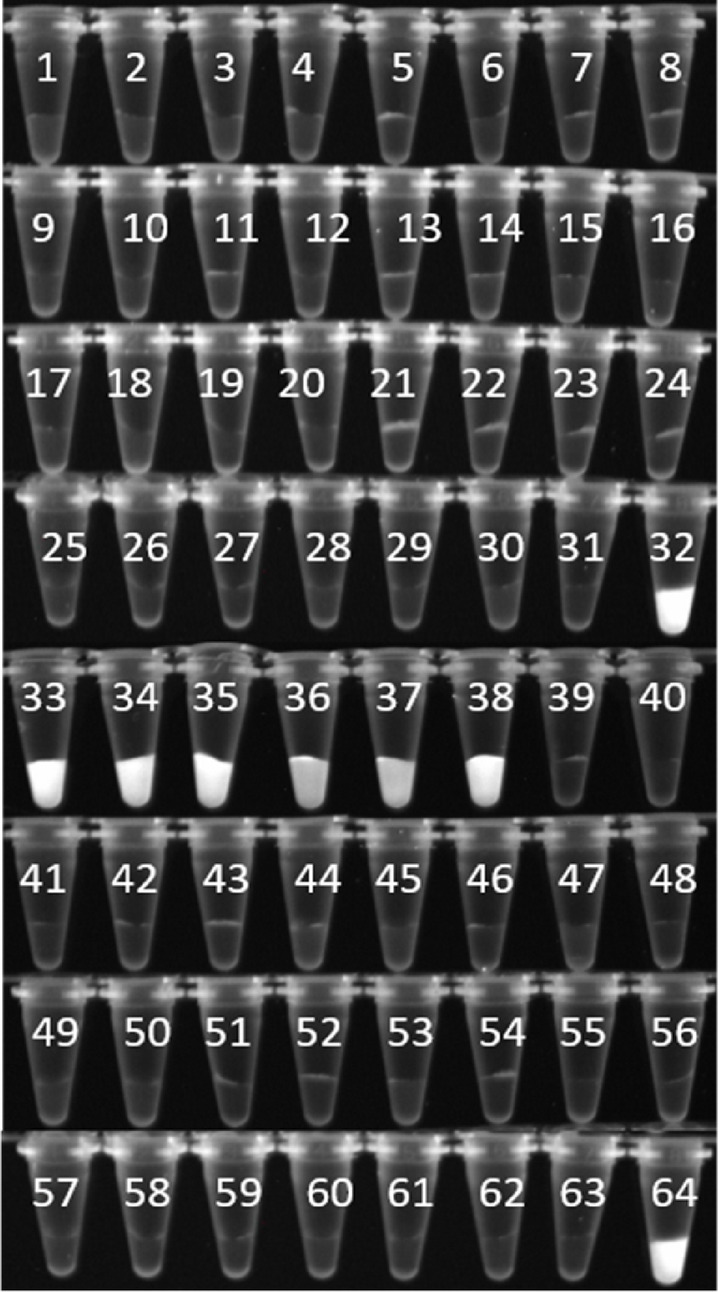
*A. palmeri + A. watsonii-*specific CRISPR-LbCas12a fluorescence assay. Sixty-four *Amaranthus* samples were preamplified using recombinase and then individually mixed with LbCas12a, the Apal+Awat_crRNA, and a quenched fluorescent reporter. After 60 min of Cas12 reaction only the expected *A. palmeri/A. watsonni* samples showed fluorescence. Numbers correspond to samples of: *A. albus* (1–4), *A. arenicola* (5–9), *A. blitum* (10–13), *A. blitoides* (14–17), *A. californicus* (18), *A. caudatus* (19–22), *A. hybridus* (23–26), *A. hypochondriacus* (27–31), *A. palmeri* (32–38), *A. powelii* (39–43), *A. retroflexus* (44–47), *A. rudis* (48), *A. spinosus* (49–52), *A. tricolor* (53), *A. tuberculatus* (54–60), *A. viridis* (61–63), *A. watsonii* (64).

Reactions left at room temperature were photographed every 5 min for 60 min. Differences in fluorescence between samples having the target sequence and samples containing polymorphisms started around 5 min and became more evident after 15 min of reaction (Supplementary Figs. 3 and 4). After 60 min of the reaction, a clear difference could be seen, where only the expected samples corresponding to *A. palmeri* and *A. watsonii* produced a strong fluorescent signal (Fig. 3).

### Quantification of fluorescence

Raw fluorescence values were obtained for 3 replicates of the 64 samples depicted in Fig. 3, after 60 min (Supplementary data 2). Three technical replicates per sample were averaged and log10-transformed, and an R-script was used for statistical analyses of the data (Supplementary Data 1). Only species where we had at least three accessions (13 species and the negative control) were used for subsequent analyses. The test for normality showed the residuals of the factors (species) were normal (Shapiro-Wilk test—*p*-value = 0.2761), but the variances were not homogeneous (Levene’s test—*p* value = 0.02693). We therefore decided to perform a Welch ANOVA, which is better suited for non-homogeneous variances and different replicate number among factors, since we had different number of accessions (biological replicates) between the species. Analysis of variance showed there was a significant difference in the fluorescence values between at least two of the species (Welch ANOVA—*p*-value < 2.2e−16), while Games-Howell post-hoc tests (Table 1) showed that the *A. palmeri* had significantly larger fluorescence than all other species and the negative controls, confirming the visual qualitative results.

**Table 1:** Games-Howell post-hoc multiple comparisons of log-10 transformed fluorescence values. Aalb (*A. albus*), Aare (*A. arenicola*), Abli (*A. blitum*), Ablit (*A. blitoides*), Acau (*A. caudatus*), Ahyb (*A. hybridus*), Ahyp (*A. hypochondriacus*), Apal (*A. palmeri*), Apow (*A. powelii*), Aret (*A. retroflexus*), Aspi (*A. spinosus*), Atub (*A tuberculatus*), Avir (*A. viridis* ), neg (negative controls). n, replicate number; Mean, mean of the log10-flourescence values; SD, Standard Deviation; GH-grouping, letters corresponding to statistically significant groupings from Games-Howell post-hoc test (*p* < 0.05). The Apal row is bolded to highlight its significantly higher mean fluorescence, as supported by the Games-Howell tests (letter “c”).

Species	*n*	Mean	SD	GH-grouping
Aalb	4	4.0969	0.0428	a
Aare	5	3.6652	0.1841	ab
Abli	4	3.8517	0.2481	ab
Ablit	4	3.6993	0.0770	b
Acau	4	3.7928	0.1014	ab
Ahyb	4	3.8594	0.2545	ab
Ahyp	5	3.8948	0.0764	b
**Apal**	**7**	**5.4734**	**0.0444**	**c**
Apow	5	3.7774	0.2670	ab
Aret	4	3.9098	0.1210	ab
Aspi	4	4.3717	0.2917	ab
Atub	7	3.7321	0.1413	b
Avir	3	3.7892	0.0753	ab
neg	3	-3.6096	0.0410	d

### Distinction between *A. palmeri* and *A. watsonii*

**Fig. 4:**
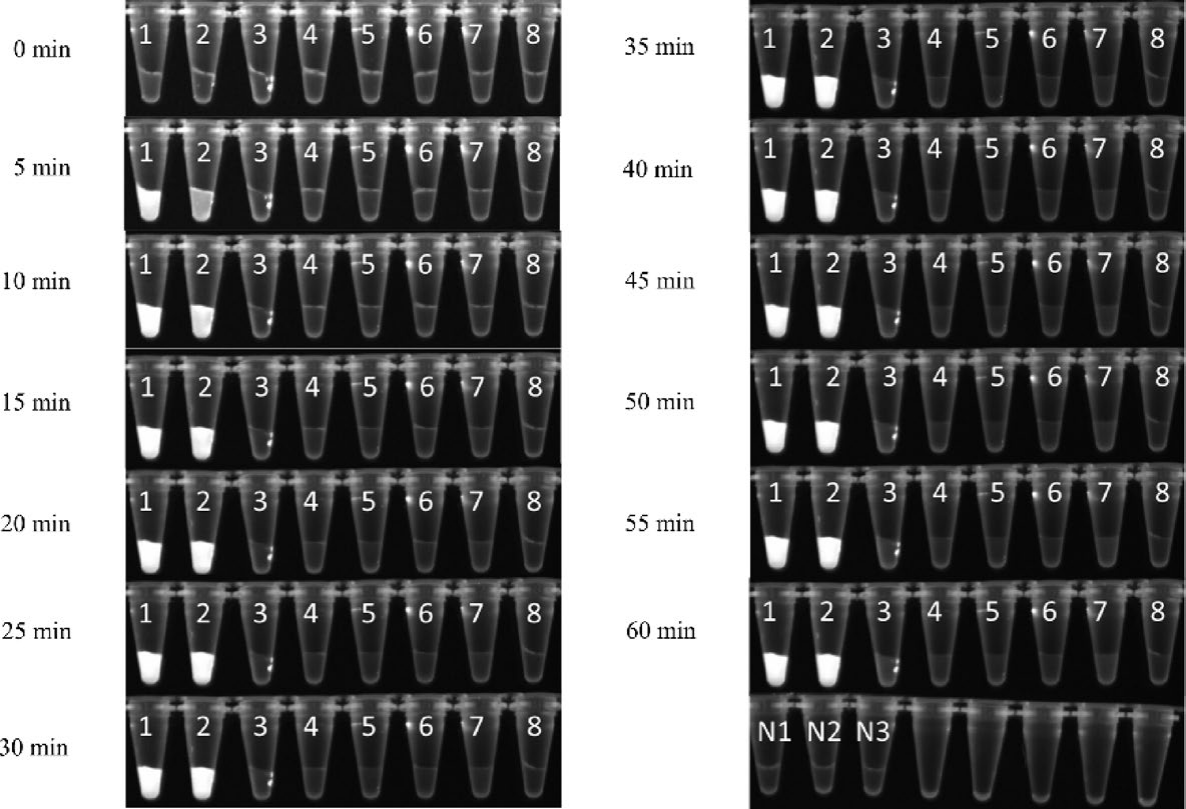
*A. watsonii-*specific CRISPR-LbCas12a 60-minute fluorescence assay. Eight accessions characterized as either *A. palmeri* or *A. watsonii* samples were pre-amplified using recombinase and then individually mixed with LbCas12a, the Apal_vs_Awat_crRNA and a quenched fluorescent reporter. *A. watsonii* samples (1–2) showed fluorescence. 3–8 = *A. palmeri* accessions, N1 = no amplicon, N2 = no amplicon or enzyme, N3= wáter.

The distinction between *A. palmeri* and *A. watsonii* based on an indel located between genes *trnS-*GAA and *rps4* was visualized by aligning six *A. palmeri* accessions and two *A. watsonii* accessions (one of these two was obtained from the USDA germplasm bank as *A. palmeri –* PI 604557, but later shown to present all genomic features of *A. watsonii*). This assay distinguishes *A. watsonii* only after samples have been pre-selected with the *A. palmeri + A. watsonii* assay. Figure 4 shows that only two tubes exhibited fluorescence. Tube no.1 belongs to *A. watsonni* accession PI 633,593, while tube No.2 corresponds to an accession initially catalogued as A. *palmeri* (PI 604557), which has shown chloroplast and nuclear polymorphisms typical of *A. watsonii* by experiments in our lab.

### Identification of *A. tuberculatus*

**Fig. 5:**
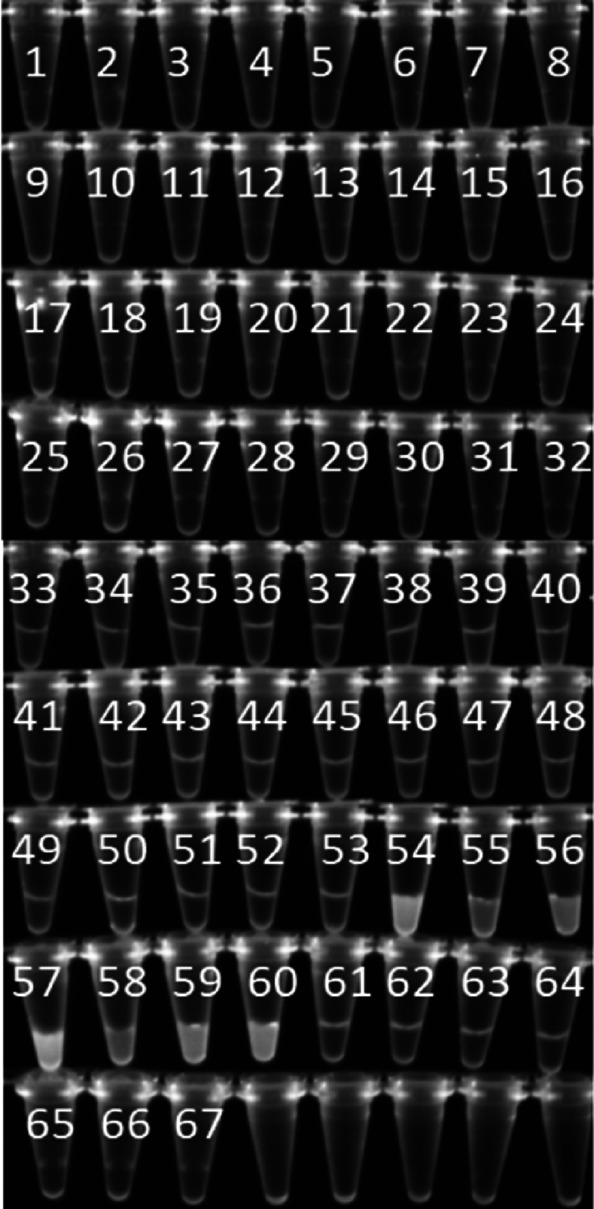
*A. tuberculatus-*specific CRISPR-LbCas12a fluorescence assay. Sixty-four *Amaranthus* samples were preamplified using recombinase and then individually mixed with LbCas12a, the Atub_crRNA and a quenched fluorescent reporter. After 60 min of Cas12 reaction only the expected *A. tuberculatus* samples showed fluorescence. Numbers correspond to samples of: *A. albus* (1–4), *A. arenicola* (5–9), *A. blitum* (10–13), *A. blitoides* (14–17), *A. californicus* (18), *A. caudatus* (19–22), *A. hybridus* (23–26), *A. hypochondriacus* (27–31), *A. palmeri* (32–38), *A. powelii* (39–43), *A. retroflexus* (44–47), *A. rudis* (48), *A. spinosus* (49–52), *A. tricolor* (53), *A. tuberculatus* (54–60), *A. viridis* (61–63), *A. watsonii* (64), negative control (65–67).

In the case of the *A. tuberculatus-*specific assay, only the samples catalogued as *A. tuberculatus* showed fluorescence after 60 min of incubation of the CRISPR-Cas12 reaction (Fig. 5), while samples from the remaining accessions corresponding to other species did not show a fluorescent signal.

### Blind sample testing

Blind sample testing was performed for 63 seed samples from the CFIA SSST, 20 samples from CEROM and 35 samples provided by our Genotyping Botany lab at CFIA in Fallowfield (Ottawa). Our three assays were run in all samples (118 in total) to distinguish three target species (*A. palmeri*,* A. watsonii* and *A. tuberculatus*) from the rest of the *Amaranthus* species samples available at our lab. Supplementary Fig. 5 shows the results after 60 min of initiating the reaction for CFIA SSST blind samples tested with the *A. palmeri + A. watsonii-*specific crRNA. A total of nine of the blind samples showed unequivocal fluorescence, indicating they corresponded to one of the two species of the assay (Supplementary Fig. 5). Further testing with the *A. watsonii-*specific test determined that one of these nine samples bound the *A. watsonii-*specific crRNA, which was not in agreement with the morphological identification performed in the Saskatoon lab (sample 57—Table 2). A re-run of the 63 samples with the *A. tuberculatus-*specific CRISPR-Cas12 test showed our assay was able to correctly match all 5 blind samples coming from CFIA-SSST (Table 2). Furthermore, out of the 118 blind samples tested, we only missed unequivocal identification in two samples: sample 57 (see above) and sample 98, which our assays could not unequivocally confirm as either *A. palmeri* or *A. watsonii* (Table 2).

**Table 2:** Identification of blind samples using CRISPR-Cas12 assays in *A. palmeri*, *A. watsonii* and *A. tuberculatus*.

Source^a^	Sample	Apal+ Awat^b^	Awat^c^	Atub^d^	CRISPR-Cas12 identification	Source identification^e^
CFIA-SSST	1*				*Amaranthus* spp.	*A. tuberculatus atypical*
CFIA-SSST	2				*Amaranthus* spp.	*A. powellii subsp. bouchonii*
CFIA-SSST	3				*Amaranthus* spp.	*A. powellii subsp. bouchonii*
CFIA-SSST	4				*Amaranthus* spp.	*A. californicus*
CFIA-SSST	5				*Amaranthus* spp.	*A. albus*
CFIA-SSST	6				*Amaranthus* spp.	*A. tricolor*
CFIA-SSST	7	x			*A. palmeri*	*A. palmeri atypical*
CFIA-SSST	8				*Amaranthus* spp.	*A. hybridus*
CFIA-SSST	9			x	*A. tuberculatus*	*A. tuberculatus*
CFIA-SSST	10				*Amaranthus* spp.	*A. tricolor*
CFIA-SSST	11			x	*A. tuberculatus*	*A. tuberculatus atypical*
CFIA-SSST	12	x			*A. palmeri*	*A. palmeri atypical*
CFIA-SSST	13	x			*A. palmeri*	*A. palmeri*
CFIA-SSST	14				*Amaranthus* spp.	*A. cruentus*
CFIA-SSST	15				*Amaranthus* spp.	*A. caudatus*
CFIA-SSST	16				*Amaranthus* spp.	*A. retroflexus*
CFIA-SSST	17				*Amaranthus* spp.	*A. spinosus*
CFIA-SSST	18				*Amaranthus* spp.	*A. powellii subsp. powellii*
CFIA-SSST	19				*Amaranthus* spp.	*A. spinosus*
CFIA-SSST	20				*Amaranthus* spp.	*A. retroflexus*
CFIA-SSST	21			x	*A. tuberculatus*	*A. tuberculatus*
CFIA-SSST	22				*Amaranthus* spp.	*A. albus atypical*
CFIA-SSST	23				*Amaranthus* spp.	*A. arenicola*
CFIA-SSST	24	x			*A. palmeri*	*A. palmeri*
CFIA-SSST	25			x	*A. tuberculatus*	*A. tuberculatus atypical*
CFIA-SSST	26				*Amaranthus* spp.	*A. cruentus*
CFIA-SSST	27				*Amaranthus* spp.	*A. hybridus*
CFIA-SSST	28				*Amaranthus* spp.	*A. powellii subsp. bouchonii*
CFIA-SSST	29				*Amaranthus* spp.	*A. arenicola*
CFIA-SSST	30				*Amaranthus* spp.	*A. albus atypical*
CFIA-SSST	31				*Amaranthus* spp.	*A. sp. (cf. powellii)*
CFIA-SSST	32				*Amaranthus* spp.	*A. retroflexus*
CFIA-SSST	33	x			*A. palmeri*	*A. palmeri*
CFIA-SSST	34				*Amaranthus* spp.	*A. caudatus*
CFIA-SSST	35			x	*A. tuberculatus*	*A. tuberculatus*
CFIA-SSST	36	x			*A. palmeri*	*A. palmeri atypical*
CFIA-SSST	37				*Amaranthus* spp.	*A. sp. (cf. powellii)*
CFIA-SSST	38				*Amaranthus* spp.	*A. sp. (cf. cruentus)*
CFIA-SSST	39				*Amaranthus* spp.	*A. albus*
CFIA-SSST	40				*Amaranthus* spp.	*A. retroflexus*
CFIA-SSST	41				*Amaranthus* spp.	*A. retroflexus atypical*
CFIA-SSST	42				*Amaranthus* spp.	*A. hybridus*
CFIA-SSST	43	x			*A. palmeri*	*A. palmeri*
CFIA-SSST	44				*Amaranthus* spp.	*A. albus atypical*
CFIA-SSST	45				*Amaranthus* spp.	*A. powellii subsp. powellii*
CFIA-SSST	46	x			*A. palmeri*	*A. palmeri*
CFIA-SSST	47				*Amaranthus* spp.	*A. retroflexus*
CFIA-SSST	48				*Amaranthus* spp.	*A. albus*
CFIA-SSST	49				*Amaranthus* spp.	*A. sp. (cf. powellii)*
CFIA-SSST	50				*Amaranthus* spp.	*A. retroflexus*
CFIA-SSST	51				*Amaranthus* spp.	*A. retroflexus atypical*
CFIA-SSST	52				*Amaranthus* spp.	*A. tricolor*
CFIA-SSST	53				*Amaranthus* spp.	*A. cruentus*
CFIA-SSST	54				*Amaranthus* spp.	*A. arenicola*
CFIA-SSST	55				*Amaranthus* spp.	*A. californicus*
CFIA-SSST	56				*Amaranthus* spp.	*A. retroflexus atypical*
CFIA-SSST	57*	x	x		*A. watsonii*	*A. palmeri atypical*
CFIA-SSST	58				*Amaranthus* spp.	*A. spinosus*
CFIA-SSST	59				*Amaranthus* spp.	*A. sp. (cf. cruentus)*
CFIA-SSST	60				*Amaranthus* spp.	*A. caudatus*
CFIA-SSST	61				*Amaranthus* spp.	*A. powellii subsp. powellii*
CFIA-SSST	62				*Amaranthus* spp.	*A. sp. (cf. cruentus)*
CFIA-SSST	63				*Amaranthus* spp.	*A. californicus*
CEROM	64			x	*A. tuberculatus*	*A. tuberculatus*
CEROM	65			x	*A. tuberculatus*	*A. tuberculatus*
CEROM	66			x	*A. tuberculatus*	*A. tuberculatus*
CEROM	67				*Amaranthus* spp.	*Hybrid A. powelli X A. retroflexus*
CEROM	68			x	*A. tuberculatus*	*A. tuberculatus*
CEROM	69				*Amaranthus* spp.	*A. powelli*
CEROM	70			x	*A. tuberculatus*	*A. tuberculatus*
CEROM	71			x	*A. tuberculatus*	*A. tuberculatus*
CEROM	72				*Amaranthus* spp.	*A. retroflexus*
CEROM	73			x	*A. tuberculatus*	*A. tuberculatus*
CEROM	74			x	*A. tuberculatus*	*A. tuberculatus*
CEROM	75			x	*A. tuberculatus*	*A. tuberculatus*
CEROM	76				*Amaranthus* spp.	*Hybrid A. powelli X A. retroflexus*
CEROM	77			x	*A. tuberculatus*	*A. tuberculatus*
CEROM	78			x	*A. tuberculatus*	*A. tuberculatus*
CEROM	79			x	*A. tuberculatus*	*A. tuberculatus*
CEROM	80			x	*A. tuberculatus*	*A. tuberculatus*
CEROM	81			x	*A. tuberculatus*	*A. tuberculatus*
CEROM	82			x	*A. tuberculatus*	*A. tuberculatus*
CEROM	83			x	*A. tuberculatus*	*A. tuberculatus*
CFIA-GB	84				*Amaranthus* spp.	*A. retroflexus*
CFIA-GB	85			x	*A. tuberculatus*	*A. tuberculatus*
CFIA-GB	86				*Amaranthus* spp.	*A. powellii*
CFIA-GB	87				*Amaranthus* spp.	*A. hybridus*
CFIA-GB	88				*Amaranthus* spp.	*A. retroflexus*
CFIA-GB	89	x			*A. palmeri*	*A. palmeri*
CFIA-GB	90	x	x		*A. watsonii*	*A. watsonii*
CFIA-GB	91				*Amaranthus* spp.	*A. cruentus*
CFIA-GB	92	x	x		*A. watsonii*	*A. palmeri or A. watsonii*
CFIA-GB	93	x			*A. palmeri*	*A. palmeri*
CFIA-GB	94				*Amaranthus* spp.	*A. retroflexus*
CFIA-GB	95			x	*A. tuberculatus*	*A. tuberculatus*
CFIA-GB	96				*Amaranthus* spp.	*A. tricolor*
CFIA-GB	97				*Amaranthus* spp.	*A. caudatus*
CFIA-GB	98*	x	x		*A. palmeri or A. watsonii*	*A. palmeri*
CFIA-GB	99				*Amaranthus* spp.	*A. retroflexus*
CFIA-GB	100	x			*A. palmeri*	*A. palmeri or A. watsonii*
CFIA-GB	101				*Amaranthus* spp.	*A. retroflexus*
CFIA-GB	102	x			*A. palmeri*	*A. palmeri*
CFIA-GB	103				*Amaranthus* spp.	*A. retroflexus*
CFIA-GB	104				*Amaranthus* spp.	*A. hypochondriacus*
CFIA-GB	105			x	*A. tuberculatus*	*A. tuberculatus*
CFIA-GB	106	x			*A. palmeri*	*A. palmeri*
CFIA-GB	107			x	*A. tuberculatus*	*A. tuberculatus*
CFIA-GB	108	x			*A. palmeri*	*A. palmeri*
CFIA-GB	109				*Amaranthus* spp.	*A. tricolor*
CFIA-GB	110				*Amaranthus* spp.	*A. powellii*
CFIA-GB	111				*Amaranthus* spp.	*A. tricolor*
CFIA-GB	112				*Amaranthus* spp.	*A. retroflexus*
CFIA-GB	113				*Amaranthus* spp.	*A. powellii*
CFIA-GB	114			x	*Amaranthus* spp.	*A. tuberculatus*
CFIA-GB	115			x	*A. tuberculatus*	*A. tuberculatus*
CFIA-GB	116			x	*A. tuberculatus*	*A. tuberculatus*
CFIA-GB	117				*Amaranthus* spp.	*A. powellii*
CFIA-GB	118	x	x		*A. watsonii*	*A. watsonii*

^a^CFIA-SSST, Canadian Food Inspection Agency Saskatoon Seed Science and Technology unit; CEROM, Centre de recherche sur les grains; CFIA-GB, Canadian Food Inspection Agency Genotyping and Botany diagnostics lab.

^b^*A. palmeri* + *A. watsonii*-specific CRISPR-Cas12 assay. An x designates a positive test.

^c^*A. watsonii* CRISPR-Cas12 assay performed on samples giving a positive result on Apal+Awat assay. An x designates a positive test.

^d^*A. tuberculatus*-specific CRISPR-Cas12 assay. An x designates a positive test.

^e^Samples marked as ‘atypical’ correspond to seeds that are less mature or outside the range of variation for typical seeds and would result in uncertain morphological identification.

*Samples were CRISPR-Cas12 test does not correspond 100% to previous morphological characterization.

### Limit of detection

**Fig. 6:**
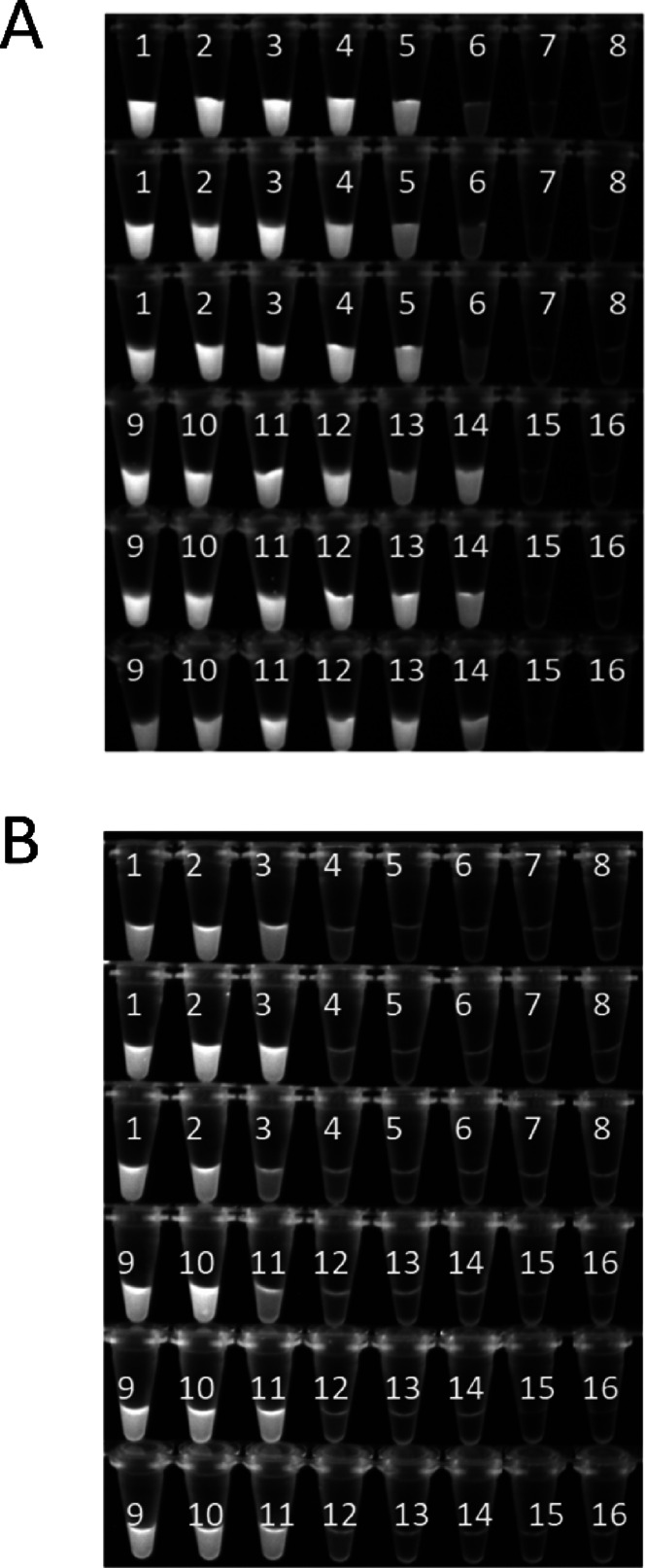
Limit of detection assay. (**A**) A recombinase reaction was performed to amplify the target region containing complementary to an *A. palmeri + A. watsonii*-specific crRNA. Five 1:10 DNA serial dilutions were used for the amplification of the first *A. palmeri* sample (Supplementary Table 1—PI633586): 1 = 15ng, 2 = 1.5ng, 3 = 0.15ng, 4 = 15pg, 5 = 1.5pg, 6 = 0.15pg, and a two controls were added: 7 = 15ng of *A. tuberculatus* (Supplementary Table 1—PI603881), and 8 = negative control without DNA. Three technical replicates were performed per run. The experiment was repeated with different *(A) palmeri* accession (Supplementary Table 1—PI549158) plus the same controls (tubes 9–16). Fluorescence was detected 60 min after the reaction was initiated. (**B**) A recombinase reaction was performed to amplify the target region containing complementary to an *A. tuberculatus*-specific crRNA. Five 1:10 DNA serial dilutions were used for the amplification of the first *A. tuberculatus* sample (Supplementary File B—PI603881): 1 = 15ng, 2 = 1.5ng, 3 = 0.15ng, 4 = 15pg, 5 = 1.5pg, 6 = 0.15pg, and a two controls were added: 7 = 15ng of an *A. palmeri* (Supplementary Table 1—PI633586), and 8 = negative control without DNA. Three technical replicates were performed per run. The experiment was repeated with different *A. tuberculatus* accession (Supplementary Table 1- PI607453) plus the respective controls (tubes 9–16). Fluorescence was detected 60 min after the reaction was initiated.

Five serial 1:10 dilutions were performed for two *A. palmeri* accessions and two *A. tuberculatus* accessions to determine the limit of detection of the assays. Fifteen nanograms were used as the starting amount since this was the optimal amount for all previous experiments. Fluorescence could be detected visually for the two *A. palmeri* accessions and the three replicates per accession up to 0.15pg when using the *A.palmeri + A. watsonii-*specific assay, while the limit of detection was weaker (0.15ng) when using the *A. tuberculatus-*specific assay (Fig. 6).

## Discussion

Being able to detect and correctly identify noxious weed seeds during trade and in the soil seed bank is important to guarantee the continuity of exports and imports, and the stability of crop production. In Canada, *A. tuberculatus* is federally regulated as a primary noxious weed seed^29^, while *A. palmeri* is not regulated. However, these plants present multiple herbicide resistance and have impacted major crops in the US like cotton, soybean and corn^3^. Previous studies have shown the establishment of *A. palmeri* populations in Japan due to imports from the US^10^. The active trade of Canada with the US, plus an expected range expansion of these species due to climate change^1,30^ are concerning for Canadian farmers and the national economy.

An increasing incorporation of novel molecular technologies that can support traditional morphological assessment for detection and identification of noxious plants is necessary to achieve an improved and streamlined process of trade. While lab-certified PCR-based technologies are now in use in laboratories around the world, improvements in efficiency, accuracy, sensitivity, and field-deployability would be desired. Both testing laboratories and farmers could benefit from technologies that can provide rapid identification of noxious weed seeds. CRISPR-Cas12 assays can be standardized with little, or no lab equipment needs, present high sensitivity, and can be completed in short time frames. This allows the technology to be transferred for testing even in the absence of molecular biology facilities.

CRISPR-Cas12 systems are starting to be used in the detection of plant pathogens, where genomic resources are plentiful to find distinct regions for targeted species^23–25,31,32^. This technology can be used for plant identification where genomic resources provide enough resolution among closely related species. In the current study with *Amaranthus*, examination of traditional plant DNA barcoding regions (e.g., *rbcL*,* matK*,* trnL*) did not show enough resolution to differentiate all the *Amaranthus* species tested. We therefore used chloroplast assemblies completed at our lab, to find regions that would provide enough polymorphisms to differentiate our target species from closely related members of the genus. Several regions that presented distinct polymorphisms were found for each of our target species, and at least two crRNAs were preliminarily tested for each target species, before selecting the crRNAs used in this study.

Additionally, in trying to optimize reagent concentration, we applied the premise from a CRISPR-Cas12 assay developed for detection of *Phytophthora ramorum* (the causal agent of sudden oak death)^24^. The *P. ramorum* study tested an array of crRNA and ssDNA reporter concentrations (40nM to 10µM) to determine the optimal values to get maximum signal. On preliminary assays we tested concentrations ranging from 50nM to 100nM for our crRNAs, and from 50nM to 1000nM for our ssDNA reporter, which resulted in optimal final concentrations of 100nM for crRNA and 100nM for the reporter. One thing to highlight is that we decided to use these concentrations for our three assays to provide a standard method for the detection of the three species. However, we note that it should be possible to try different concentrations to increase the fluorescent signal we obtained with our *A. tuberculatus-*specific test (Fig. 5). Moreover, a preliminary test performed for detection of *A. tuberculatus* provided a stronger visual signal on the testing panel (Supplementary Fig. 6A) but failed to detect two *A. tuberculatus* blind samples (Supplementary Fig. 6B) and therefore was not further implemented in the current study. Sequencing of the false negative *A. tuberculatus* samples revealed a mutation on the crRNA binding site (Supplementary Fig. 6C) supporting why this test was not effective. Further, potential modifications to increase the fluorescent signal when standardizing a CRISPR-Cas12 detection include: changes in the reaction buffer, additives (e.g., PEG, BSA), and 3’ and 5’ crRNA extensions^33^. We are planning to test these modifications in future studies.

To our knowledge, the only other report of CRISPR-Cas12 technology used to distinguish morphologically similar plants was done in the genus *Phyllantus*, a group of herbal plants used in commercialized products^26^. The CRISPR-Cas12 assay in *Phyllanthus* used only three additional species (*P. urinaria*,* P. debilis*,* P. virgatus*) to assess the specificity of the assay to their target species (*P. amarus*). From these three additional species, only *P. debilis* is relatively close to *P. amarus* within the genus^34,35^. This could result in cross-reactivity and false positives if species that are more closely related to *P. amarus* were to be tested. Our CRISPR-Cas12 assays initially used 28 accessions corresponding to 15 species to validate the accuracy of the test and anticipate potential cross-reactivity of the test with closely related species of the *Amaranthus* genus. While the *Phyllanthus* assay showed similar sensitivity in its limit of detection to our *A. palmeri* + *A. watsonii* CRISPR-Cas12 test (0.8pg vs. 0.15pg, respectively), the accuracy of our assays seems superior. The *Phyllanthus* assay highlighted 90% accuracy to detect *P. amarus* in 20 blind samples, with two *P. reticulatus* showing as false positives (Fig. 4 in reference: ^26^); this latter species does not seem to be closely related to *P. amarus*^35,36^. In the meantime, our CRISPR-Cas12 assay for *A. palmeri + A. watsonii* and for *A. tuberculatus* were 100% accurate with no false positives when testing 118 blind samples (Table 2); and our serial *A. watsonii* test performed after our *A. palmeri + A. watsonii* assay only failed to correctly classify a sample as definitely *A. watsonii* in 2 out of 19 samples pre-classified as either *A. palmeri* or *A. watsonii.* All our tests presented multiple species from the same clade and were able to distinguish our three target species from their respective closest related species. We base this conclusion on the most complete reported phylogeny of the genus^4^. In terms of the time needed to complete our assay, we established 1 h as the time to check the results from our three tests, but depending on the test, a clear distinction of positive tests can be established as quickly as 5 min in the case of the *A. watsonii-*specific assay (Fig. 4). The CRISPR-Cas12 established for *Phyllantus* used two hours for their detection^26^, while other CRISPR-Cas tests for detection of GMO genes have used close to 30 min as their standard time for detection^23^.

In plants, CRISPR-Cas12 has also been used to detect presence/absence of larger genomic regions. For example, the detection of a GMO gene in rice (*Cry1c*) was tested with different crRNAs^23^. Their assay limit of resolution was tested down to 1pg of purified DNA, while in comparison, our test can use 0.15pg of DNA. A similar approach has been used to detect GM soybean using the CRISPR-Cas12 system to target the *CaMV35s* promoter of the transgene^22^. While the assay for GM soybean used a Loop-mediated Isothermal Amplification (LAMP) as part of their field-deployable strategy, we decided to use recombinase in our assay development for its simplicity to achieve amplification of the target. In general, 6 primers are needed for LAMP vs. 2 for recombinase, making standardization of the LAMP reaction cumbersome. The advantage of isothermal approaches is that they can be completed without the need for a thermocycler. We are currently working on a fast field-deployable DNA extraction protocol to make sure our assay can be done from beginning to end without needing any lab equipment.

Besides CRISPR-Cas12, other Cas enzymes have also been used for genotyping. For example, a CRISPR-Cas9 system was used to genotype wheat resistance alleles to stripe rust, the presence of herbicide-resistant alleles in rice, and transgenic elements in both rice and tobacco plants^37^. In this study, the researchers used CRISPR-Cas9 to cut FAM-labelled amplicons containing the target polymorphic region, so that when the ribonucleoprotein complex of Cas9 and guide RNA found the target region, the fluorophore-bound cut fragment could interact with an anti-FAM antibody region in a lateral flow assay strip. While Cas9 does not present collateral activity, the researchers of this study also used pre-amplification of the target region to increase sensitivity of their assay, similarly to what we presented in the current study. Their assay was highly accurate in detecting their targeted genes and gene mutations. One factor that helped achieve specificity in both our assay and the assay to distinguish resistance wheat alleles^37^, was the presence of indels/mutations in proximity of the PAM regions for Cas12 and Cas9, respectively. Mutations within or in the proximity of the PAM regions are known to provide higher specificity for both enzymes^37–40^. Another report used Cas13 to characterize the presence of a glyphosate resistance gene in soybean^41^. The use of Cas13 has the advantage of not needing a PAM site, therefore allowing to target polymorphisms anywhere. However, Cas 13 targets RNA molecules, so the targeted gene must be actively transcribed. In our case, since we aim to find DNA polymorphisms, we used Cas12, but using Cas13 could be a good alternative in future studies where we want to determine the viability of a noxious weed. This is because gene expression is a proxy for metabolic activity, while non-viable tissues can still carry intact DNA. In this case, a constitutively expressed gene with enough polymorphic resolution could have a dual role as a barcode and viability marker.

Finally, other non-Cas molecular methods have been standardized to distinguish between closely related *Amaranthus* species. Using Genotyping By Sequencing, a KASP (Kompetitive Allele-Specific PCR) assay was designed to detect a single seed of *A. palmeri* mixed at 0.5 and 1% proportions in *A. tuberculatus* seeds, and to distinguish individual seeds of *A. palmeri* from 8 *Amaranthus* species^42^. Their test for individual samples provided high accuracy > 99% for three different markers in a large panel (1248 individuals of *A. palmeri* and other species). Our experiment provided 100% accuracy when identifying members of the *A. palmeri + A. watsonii* clade (19 out of 19 individuals correctly identified in Table 2). When using our CRISPR-Cas12 test to distinguish *A. palmeri* from *A. watsonii* samples in these 19 individuals, sample 57 was identified as *A. watsonii*, while the morphological identification was *A. palmeri*, and the tests were inconclusive in determining if sample 98 was *A. palmeri* or *A. watsonii.* We speculate that factors like hybridization or morphological misidentification, could be among the reasons why these two samples did not perfectly match morphological identification. We recognize our panel is less robust than the panel used by Brusa et al., ^42^, but our assays provide an additional tool with more *Amaranthus* species tested. A second assay using qPCR on a polymorphic ITS region was previously used to distinguish *A. palmeri* from other *Amaranthus* species^43^. The specificity of this assay was based on primers that spanned *A. palmeri* polymorphisms, with a cluster analysis supporting no overlap between species when the qPCR test was performed for *A. palmeri*,* A. tuberculatus*,* A. arenicola* and negative samples. A second manuscript using the same primers was used to identify Palmer Amaranth individually or in seed mixes from 15 other species and hybrid accessions^44^. In this article additional markers were evaluated to increase resolution, which allowed distinguishing Palmer amaranth from *A. dubious* and *A. spinosus* but not from *A. watsonii.* Older molecular technologies have also been used in the past to separate *Amaranthus* species, including traditional barcoding and PCR-RFLP, but these methods were limited in the number of species tested and usually required longer times to achieve results^45,46^. Comparatively with these techniques, our assays offer a rapid way of qualitative assessment for species identification with little need for lab equipment.

In conclusion, we have standardized three assays to distinguish *A. palmeri*,* A. watsonii* and *A. tuberculatus* from closely related *Amaranthus* species, which could support the identification of these noxious weed seeds in the regulatory framework and in agricultural settings. For example, during morphological detection and identification of contaminant seeds mixed with trading commodities, the species of *Amaranthus* can’t be identified sometimes due to plasticity in the genus. In these cases, our assay would support accurate identification of the noxious weed. We realize that testing was done in a limited number of species and accessions. If the test is to be implemented in another setting or replicated to test new samples in a different laboratory, we recommend performing sequencing of potential false positives or negatives if the results conflict with the morphological identification. Likewise, when testing alternative primers or crRNAs a verification of the complete pipeline with samples that have been clearly identified to species, will be necessary.

Since the limit of detection of our assays is the picogram scale, the method could also be implemented in situations where the target weeds are mixed with crop seed or other *Amaranthus* seeds, as has been done before with other assays^42,44,47^. We recognize that further testing will be necessary to see how the limit of detection may be affected by running a pipeline of amplification and CRISPR-Cas12 in a seed mix where the target weed is diluted.

Our assays do not need any lab equipment once DNA is extracted and can be completed in less than an hour after DNA is available. However, we realize that for this assay to be fully transferable for field deployment or for labs without molecular biology equipment, we need to add a rapid equipment-less DNA extraction step.

Finally, to further decrease the likelihood of cross-contamination, we are aiming to incorporate the amplification and CRISPR-Cas12 reaction in a single tube (one-pot reaction), that can maintain high efficiency and high fluorescent signal. Different mechanisms are available for inhibiting the CRISPR-Cas12 reaction until sufficient amplicon is present to avoid low reaction efficiency^48,49^.

## Supplementary Information

Below is the link to the electronic supplementary material.

Supplementary Material 1

## Data Availability

All data generated or analysed during this study are included in this published article (and its Supplementary Information files).

## References

[1] Oliveira, M. C. et al. Palmer Amaranth (*Amaranthus palmeri*) adaptation to US midwest agroecosystems. *Front. Plant Sci.* **4**, 887629. 10.3389/fagro.2022.887629 (2022).

[2] Beckie, H. J. Herbicide-resistant weed management: Focus on glyphosate. *Pest Manag. Sci.* **67**, 1037–1048. 10.1002/ps.2195 (2011). 21548004 10.1002/ps.2195

[3] Ward, S. M., Webster, T. M. & Steckel, L. E. Palmer Amaranth (*Amaranthus palmeri*): A review. *Weed Technol.* **27**, 12–27. 10.1614/wt-d-12-00113.1 (2013).

[4] Waselkov, K. E., Boleda, A. S. & Olsen, K. M. A phylogeny of the genus *Amaranthus* (Amaranthaceae) Based on several low-copy nuclear loci and chloroplast regions. *Syst. Bot.* **43**, 439–458. 10.1600/036364418X697193 (2018).

[5] Amaranthus palmeri S.Watson. *EPPO Bull.* **50**, 535–542. 10.1111/EPP.12715 (2020).

[6] Massinga, R., Currie, R., Horak, M. & Boyer, J. Interference of Palmer amaranth in corn. *Weed Sci.* **49**, 202–208. 10.1614/0043-1745(2001)049[0202:IOPAIC]2.0.CO;2 (2001).

[7] Bensch, C. N., Horak, M. J. & Peterson, D. Interference of redroot pigweed (*Amaranthus retroflexus*), Palmer amaranth (*A. palmeri*), and common waterhemp *(A. rudis*) in soybean. *Weed Sci.* **51**, 37–43. 10.1614/0043-1745(2003)051 (2003). [0037:IORPAR]2.0.CO;2.

[8] Tranel, P. J. Herbicide resistance in *Amaranthus tuberculatus* †. *Pest Manag. Sci.* **77**, 43–54. 10.1002/PS.6048 (2021). 32815250 10.1002/ps.6048

[9] Xu, H. et al. Species identification, phylogenetic analysis and detection of herbicide-resistant biotypes of *Amaranthus* based on ALS and ITS. *Sci. Rep.* **10**, 1–9. 10.1038/s41598-020-68541-x (2020). 32678146 10.1038/s41598-020-68541-x PMC7366686

[10] Shimono, A. et al. Initial invasion of glyphosate-resistant *Amaranthus palmeri* around grain-import ports in Japan. *Plants People Planet.* **2**, 640–648. 10.1002/ppp3.10156 (2020).

[11] Sun, S. *China’s ban on Canadian Canola: reasons, impacts and policy perspectives* (University of Alberta, 2020).

[12] Costea, M., Weaver, S. E. & Tardif, F. J. The biology of Canadian weeds. 130. Amaranthus retroflexus L., A. powellii S. Watson and A. hybridus L. *Can. J. Plant Sci.* **84**, 631–668. 10.4141/p02-183 (2004).

[13] Nidhi, S. et al. Novel CRISPR-Cas systems: An updated review of the current achievements, applications, and future research perspectives. *Int. J. Mol. Sci.* **22**, 3327. 10.3390/ijms22073327 (2021). 33805113 10.3390/ijms22073327 PMC8036902

[14] Li, S. Y. et al. CRISPR-Cas12a has both cis- and trans-cleavage activities on single-stranded DNA. *Cell Res.* **28**, 491–493. 10.1038/s41422-018-0022-x (2018). 29531313 10.1038/s41422-018-0022-x PMC5939048

[15] Li, S. Y. et al. CRISPR-Cas12a-assisted nucleic acid detection. *Cell. Discov.*. **4**, 20. 10.1038/s41421-018-0028-z (2018). 29707234 10.1038/s41421-018-0028-z PMC5913299

[16] Chen, J. S. et al. CRISPR-Cas12a target binding unleashes indiscriminate single-stranded DNase activity. *Science*. **360**, 436–436. 10.1126/SCIENCE.AAR6245 (2018). 10.1126/science.aar6245 PMC6628903 29449511

[17] Gootenberg, J. S. et al. Nucleic acid detection with CRISPR-Cas13a/C2c2. *Science*. **356**, 438–438. 10.1126/SCIENCE.AAM9321 (2017). 10.1126/science.aam9321 PMC5526198 28408723

[18] Chaijarasphong, T., Thammachai, T., Itsathitphaisarn, O., Sritunyalucksana, K. & Suebsing, R. Potential application of CRISPR-Cas12a fluorescence assay coupled with rapid nucleic acid amplification for detection of white spot syndrome virus in shrimp. *Aquaculture* **512**, 734340–734340. 10.1016/J.AQUACULTURE.2019.734340 (2019).

[19] Ali, Z. et al. iSCAN: An RT-LAMP-coupled CRISPR-Cas12 module for rapid, sensitive detection of SARS-CoV-2. *Virus Res.* **288**, 198129. 10.1016/j.virusres.2020.198129 (2020). 32822689 10.1016/j.virusres.2020.198129 PMC7434412

[20] Broughton, J. P. et al. CRISPR–Cas12-based detection of SARS-CoV-2. *Nat. Biotechnol.* **38** (7), 870–874, (2020). 10.1038/s41587-020-0513-4 10.1038/s41587-020-0513-4 PMC9107629 32300245

[21] Liu, H. et al. RPA-Cas12a-FS: A frontline nucleic acid rapid detection system for food safety based on CRISPR-Cas12a combined with recombinase polymerase amplification. *Food Chem.* **334**, 127608. 10.1016/j.foodchem.2020.127608 (2021). 32711280 10.1016/j.foodchem.2020.127608

[22] Wu, H., He, J. S., Zhang, F., Ping, J. & Wu, J. Contamination-free visual detection of CaMV35S promoter amplicon using CRISPR/Cas12a coupled with a designed reaction vessel: Rapid, specific and sensitive. *Anal. Chim. Acta*. **1096**, 130–137. 10.1016/J.ACA.2019.10.042 (2020). 31883579 10.1016/j.aca.2019.10.042

[23] Zhang, Y. M., Zhang, Y. & Xie, K. Evaluation of CRISPR/Cas12a-based DNA detection for fast pathogen diagnosis and GMO test in rice. *Mol. Breeding*. **40**, 1–12. 10.1007/S11032-019-1092-2/FIGURES/4 (2020).

[24] Guo, Y. et al. CRISPR/Cas12a-based approaches for efficient and accurate detection of *Phytophthora ramorum*. *Front. Cell. Infect. Microbiol.* **13**, 1218105. 10.3389/fcimb.2023.1218105 (2023). 37441240 10.3389/fcimb.2023.1218105 PMC10333691

[25] Zhang, A. et al. CRISPR/Cas12a Coupled with recombinase polymerase amplification for sensitive and specific detection of *Aphelenchoides besseyi*. *Front. Bioeng. Biotechnol.* **10**, 912959. 10.3389/fbioe.2022.912959 (2022). 35845427 10.3389/fbioe.2022.912959 PMC9279656

[26] Buddhachat, K. et al. Bar-cas12a, a novel and rapid method for plant species authentication in case of *Phyllanthus amarus* Schumach. & Thonn. *Sci. Rep.* **11**, 20888. 10.1038/s41598-021-00006-1 (2021). 34686666 10.1038/s41598-021-00006-1 PMC8536675

[27] Raiyemo, D. A. & Tranel, P. J. Comparative analysis of dioecious *Amaranthus* plastomes and phylogenomic implications within Amaranthaceae s.s. *BMC Ecol. Evol.* **23**10.1186/s12862-023-02121-1 (2023). 10.1186/s12862-023-02121-1 PMC10164334 37149567

[28] Nguyen, L. T., Smith, B. M. & Jain, P. K. Enhancement of trans-cleavage activity of Cas12a with engineered crRNA enables amplified nucleic acid detection. *Nat. Commun.* **11**, 1–13 (2020). 10.1038/s41467-020-18615-1 10.1038/s41467-020-18615-1 PMC7528031 32999292

[29] CFIA. *Weed seeds order*, https://laws-lois.justice.gc.ca/eng/regulations/SOR-2016-93/FullText.html(2016).

[30] USDA. *Weed risk assessment for Amaranthus palmeri (Amaranthaceae)—Palmer’s amaranth* (USDA, 2020).

[31] Aman, R. et al. Rapid, and sensitive detection of plant RNA viruses with one-pot RT-RPA–CRISPR/Cas12a assay. *Front. Microbiol.* **11**, 610872. 10.3389/fmicb.2020.610872 (2020). 33391239 10.3389/fmicb.2020.610872 PMC7773598

[32] Anbazhagan, P. et al. Advances in plant pathogen detection: integrating recombinase polymerase amplification with CRISPR/Cas systems. *3 Biotech.* **14** (214). 10.1007/s13205-024-04055-x (2024). 10.1007/s13205-024-04055-x PMC11349965 39211481

[33] Qiu, M., Zhou, X. M. & Liu, L. Improved strategies for CRISPR-Cas12-based nucleic acids detection. *J. Anal. Test.* **6**, 44–52. 10.1007/s41664-022-00212-4 (2022). 35251748 10.1007/s41664-022-00212-4 PMC8883004

[34] Bouman, R. W. et al. Molecular phylogenetics of Phyllanthus sensu lato (Phyllanthaceae): Towards coherent monophyletic taxa. *TAXON* **70**, 72–98. 10.1002/tax.12424 (2021).

[35] Kathriarachchi, H. et al. Phylogenetics of tribe Phyllantheae (Phyllanthaceae; Euphorbiaceae sensu lato) based on nrITS and plastid matK DNA sequence data. *Am. J. Bot.* **93**, 637–655. 10.3732/ajb.93.4.637 (2006). 21646224 10.3732/ajb.93.4.637

[36] Kiran, K. R. et al. Untargeted metabolomics and DNA barcoding for discrimination of *Phyllanthus* species. *J. Ethnopharmacol.* **273**, 113928. 10.1016/j.jep.2021.113928 (2021). 33631274 10.1016/j.jep.2021.113928

[37] Sánchez, E., Ali, Z., Islam, T. & Mahfouz, M. A CRISPR-based lateral flow assay for plant genotyping and pathogen diagnostics. *Plant. Biotechnol. J.* **20**, 2418–2429. 10.1111/pbi.13924 (2022). 36072993 10.1111/pbi.13924 PMC9674313

[38] Zheng, T. et al. Profiling single-guide RNA specificity reveals a mismatch sensitive core sequence. *Sci. Rep.* **7**, 40638. 10.1038/srep40638 (2017). 28098181 10.1038/srep40638 PMC5241822

[39] Anderson, E. M. et al. Systematic analysis of CRISPR–Cas9 mismatch tolerance reveals low levels of off-target activity. *J. Biotechnol.* **211**, 56–65. 10.1016/j.jbiotec.2015.06.427 (2015). 26189696 10.1016/j.jbiotec.2015.06.427

[40] Li, S. Y. et al. CRISPR-Cas12a-assisted nucleic acid detection. *Cell Discov.* **4**, 1–4. 10.1038/s41421-018-0028-z (2018). 10.1038/s41421-018-0028-z PMC5913299 29707234

[41] Abudayyeh, O. O., Gootenberg, J. S., Kellner, M. J. & Zhang, F. Nucleic acid detection of plant genes using CRISPR-Cas13. *Crispr J.* **2**, 165–171. 10.1089/crispr.2019.0011 (2019). 31225754 10.1089/crispr.2019.0011 PMC7001462

[42] Brusa, A. et al. A needle in a seedstack: An improved method for detection of rare alleles in bulk seed testing through KASP. *Pest Manag. Sci.* **77**, 2477–2484. 10.1002/ps.6278 (2021). 33442897 10.1002/ps.6278

[43] Murphy, B. P., Plewa, D. E., Phillippi, E., Bissonnette, S. M. & Tranel, P. J. A quantitative assay for *Amaranthus palmeri* identification. *Pest Manag. Sci.* **73**, 2221–2224. 10.1002/PS.4632 (2017). 28580655 10.1002/ps.4632

[44] Bratsch, S. et al. Optimizing Palmer amaranth (*Amaranthus palmeri*) genetic testing of seeds using real-time (quantitative) PCR. *Weed Sci.* **72**, 732–739. 10.1017/wsc.2024.58 (2024).

[45] Park, Y. J. & Nishikawa, T. Rapid identification of *Amaranthus caudatus* and *Amaranthus hypochondriacus* by sequencing and PCR-RFLP analysis of two starch synthase genes. *Genome* **55**, 623–628. 10.1139/g2012-050 (2012). 22892013 10.1139/g2012-050

[46] Wetzel, D. K., Horak, M. J. & Skinner, D. Z. Use of PCR-based molecular markers to identify weedy *Amaranthus* species. *Weed Sci.* **47**, 518–523. 10.1017/S0043174500092201 (1999).

[47] Murphy, B. P. & Tranel, P. J. Identification and validation of *Amaranthus* species-specific SNPs within the ITS region: Applications in quantitative species identification. *Crop Sci.* **58**, 304–311. 10.2135/cropsci2017.06.0359 (2018).

[48] Chen, Y. et al. Photoactivatable CRISPR/Cas12a strategy for one-pot DETECTR molecular diagnosis. *Anal. Chem.* **94**, 9724–9731. 10.1021/acs.analchem.2c01193 (2022). 35762828 10.1021/acs.analchem.2c01193

[49] Cho, E., Yun, D. & Jung, C. One-pot RPA/CRISPR-Cas12a assay with photomodulated aptamer-based inhibitors. *Sens. Actuators B*. **412**, 135790. 10.1016/j.snb.2024.135790 (2024).

