# Supplementary material for: CRISPR-Cas12a fluorescence assays identify weedy Amaranthus species
Source: Sci Rep. 2026 Apr 28;16:20114. doi: 10.1038/s41598-026-49454-7 (PMC13324167; doi:10.1038/s41598-026-49454-7)
Supplement: Supplementary file 1 — Supplementary Material 1 [file 41598_2026_49454_MOESM1_ESM.pdf]

CRISPR-Cas12a fluorescence assays identify weedy *Amaranthus* species

Leonardo Galindo-González[1*] and Andrée Ann Dupras[1]

Supplementary Tables

Supplementary Table 1. Plant samples used in the different experiments. The MIRL ID refers to the internal CFIA Molecular Identification Research Lab ID. The GRIN ID corresponds to the USDA National Plant Germplasm System ID (https://npgsweb.ars-grin.gov/gringlobal/search).

MIRL ID	Genus	Species	Origin	GRIN ID	Comment
MIRL22-Aalb-01	Amaranthus	albus	Canada, Saskatoon	PI633580	received from USDA
MIRL22-Aalb-02	Amaranthus	albus	Harrow	N/A	received from collaborators at AAFC
MIRL23-Aalb-07	Amaranthus	albus	United States, North Carolina	PI632244	received from USDA
MIRL23-Aalb-04	Amaranthus	albus	United States, Washington	PI654389	received from USDA
MIRL22-Aare-01	Amaranthus	arenicola	United States, Kansas	PI599670	received from USDA
MIRL23-Aare-02	Amaranthus	arenicola	United States, Kansas	PI599671	received from USDA
MIRL23-Aare-04	Amaranthus	arenicola	United States, Kansas	PI607459	received from USDA
MIRL23-Aare-05	Amaranthus	arenicola	United States, Texas	PI667168	received from USDA
MIRL23-Aare-03	Amaranthus	arenicola	United States, Kansas	PI 599673	received from USDA
MIRL22-Ablit-04	Amaranthus	blitoides	Canada	PI608663	received from USDA
MIRL22-Ablit-03	Amaranthus	blitoides	Harrow	N/A	received from collaborators at AAFC
MIRL23-Ablit-06	Amaranthus	blitoides	United States, Utah	PI 612854	received from USDA
MIRL23-Ablit-07	Amaranthus	blitoides	United States, New Mexico	PI 690354	received from USDA
MIRL23-Abli-07	Amaranthus	blitum	United States, North Carolina	PI632245	received from USDA
MIRL22-Abli-01	Amaranthus	blitum	United States, North Carolina	PI632245	received from USDA
MIRL23-Abli-05	Amaranthus	blitum	United States, Florida	AMES 34071	received from USDA
MIRL23-Abli-06	Amaranthus	blitum	United States, District of Columbia	PI 612860	received from USDA
MIRL23-Acal-02	Amaranthus	californicus	United States, California	PI595319	received from USDA
MIRL23-Acau-09	Amaranthus	caudatus	United States, California	PI690570	received from USDA
MIRL22-Acau-03	Amaranthus	caudatus	United States, New Jersey	AMES 5687	received from USDA
MIRL23-Acau-05	Amaranthus	caudatus	United States, New Jersey	PI553073	received from USDA
MIRL23-Acau-06	Amaranthus	caudatus	United States, Iowa	PI 632249	received from USDA
MIRL22-Ahyb-03	Amaranthus	hybridus	Harrow	N/A	received from collaborators at AAFC
MIRL23-Ahyb-06	Amaranthus	hybridus	United States, Indiana	PI603895	received from USDA
MIRL23-Ahyb-05	Amaranthus	hybridus	Puerto Rico	AMES 5152	received from USDA
MIRL22-Ahyb-04	Amaranthus	hybrid	Ontario	AMES 14944	received from USDA
MIRL22-Ahyp-01	Amaranthus	hypochondriacus	Iowa	PI658730	received from USDA
MIRL23-Ahyp-17	Amaranthus	hypochondriacus	Mexico, Chihuahua	PI633589	received from USDA
MIRL23-Ahyp-15	Amaranthus	hypochondriacus	Mexico, Puebla	PI604584	received from USDA
MIRL23-Ahyp-14	Amaranthus	hypochondriacus	Mexico, Sonora	PI599682	received from USDA
MIRL23-Ahyp-11	Amaranthus	hypochondriacus	United States, Missouri	PI698341	received from USDA
MIRL22-Apal-11	Amaranthus	palmeri	United States, Arizona	PI686461	received from USDA
MIRL22-Apal-07	Amaranthus	palmeri	Dakar	PI633586	received from USDA
MIRL22-Apal-06	Amaranthus	palmeri	Dakar	PI633587	received from USDA
MIRL22-Apal-09	Amaranthus	palmeri	Mali	PI549158	received from USDA
MIRL22-Apal-08	Amaranthus	palmeri	Mexico, Puebla	PI604557	received from USDA
MIRL22-Apal-10	Amaranthus	palmeri	Mexico, Veracruz	PI667167	received from USDA
MIRL22-Apal-12	Amaranthus	palmeri	United States, Arizona	PI632236	received from USDA
MIRL22-Apow-02	Amaranthus	powelli sub. Powelli	United States, Maine	AMES 29205	received from USDA
MIRL22-Apow-03	Amaranthus	powellii	Harrow	N/A	received from collaborators at AAFC
MIRL23-Apow-06	Amaranthus	powellii sub. Powellii	United States, New Mexico	PI649309	received from USDA
MIRL23-Apow-05	Amaranthus	powellii sub. Powellii	United States, Texas	PI632241	received from USDA
MIRL22-Apow-04	Amaranthus	powellii	unknown	N/A	CFIA internal sample
MIRL22-Aret-08	Amaranthus	retroflexus	Canada	AMES 5328	received from USDA
MIRL22-Aret-09	Amaranthus	retroflexus	Harrow	N/A	received from collaborators at AAFC
MIRL23-Aret-12	Amaranthus	retroflexus	Ontario	AMES 35199	received from USDA
MIRL23-Aret-15	Amaranthus	retroflexus	Utah	PI612857	received from USDA
MIRL22-Arud-01	Amaranthus	rudis	Harrow	N/A	received from collaborators at AAFC
MIRL22-Aspi-01	Amaranthus	spinosus	United States, North Carolina	PI632248	received from USDA
MIRL23-Aspi-04	Amaranthus	spinosus	Cuba, La Habana	PI642740	received from USDA
MIRL22-Aspi-02	Amaranthus	spinosus	Harrow	N/A	received from collaborators at AAFC
MIRL23-Aspi-03	Amaranthus	spinosus	United States, North Carolina	PI 632248	received from USDA
MIRL22-Atri-05	Amaranthus	spinosus	United States, North Carolina	PI632248	received from USDA
MIRL23-Atub-24	Amaranthus	tricolor	United States, New Jersey	AMES 5303	received from USDA
MIRL23-Atub-28	Amaranthus	tuberculatus	United States, Illinois	PI603864	received from USDA
MIRL22-Atub-17	Amaranthus	tuberculatus	United States, Indiana	PI603881	received from USDA
MIRL22-Atub-20	Amaranthus	tuberculatus	United States, Iowa	PI553086	received from USDA
MIRL22-Atub-19	Amaranthus	tuberculatus	United States, Iowa	PI604247	received from USDA
MIRL22-Atub-18	Amaranthus	tuberculatus	United States, Kansas	PI607453	received from USDA
MIRL23-Atub-26	Amaranthus	tuberculatus	United States, Nebraska	PI603873	received from USDA
MIRL22-Avir-01	Amaranthus	viridis	United States, Florida	PI654388	received from USDA
MIRL23-Avir-02	Amaranthus	viridis	Puerto Rico	AMES 5150	received from USDA
MIRL23-Avir-03	Amaranthus	viridis	United States, Florida	PI 654388	received from USDA
MIRL22-Awat-01	Amaranthus	watsonii	Mexico, Colima	PI633593	received from USDA

Supplementary Figures

Supplementary Figure 1. Chloroplast genome alignment between genes *trnS*-GAA and *rps4*. The section was selected to distinguish *A. palmeri* from *A. watsonii* using a *A. watsonii*-specific crRNA. The crRNA (boxed) starts with a PAM sequence needed for Cas 12 binding (TTTC). Accession PI 604557 has been characterized in our lab as *A. watsonii.*

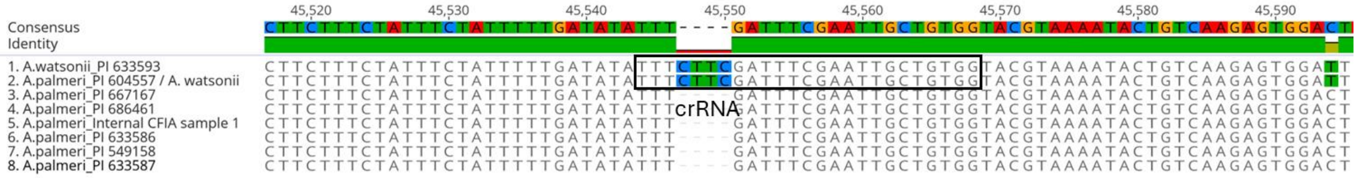

Supplementary Figure 2. Chloroplast genome alignment between genes *trnG*-GCC and *trnFm*-CAU where an *A. tuberculatus*-specific crRNA was designed*.* The alignment includes 28 chloroplast genomes previously assembled in our laboratory plus genomes used in a previous study [Raiyemo and Tranel, 2023].

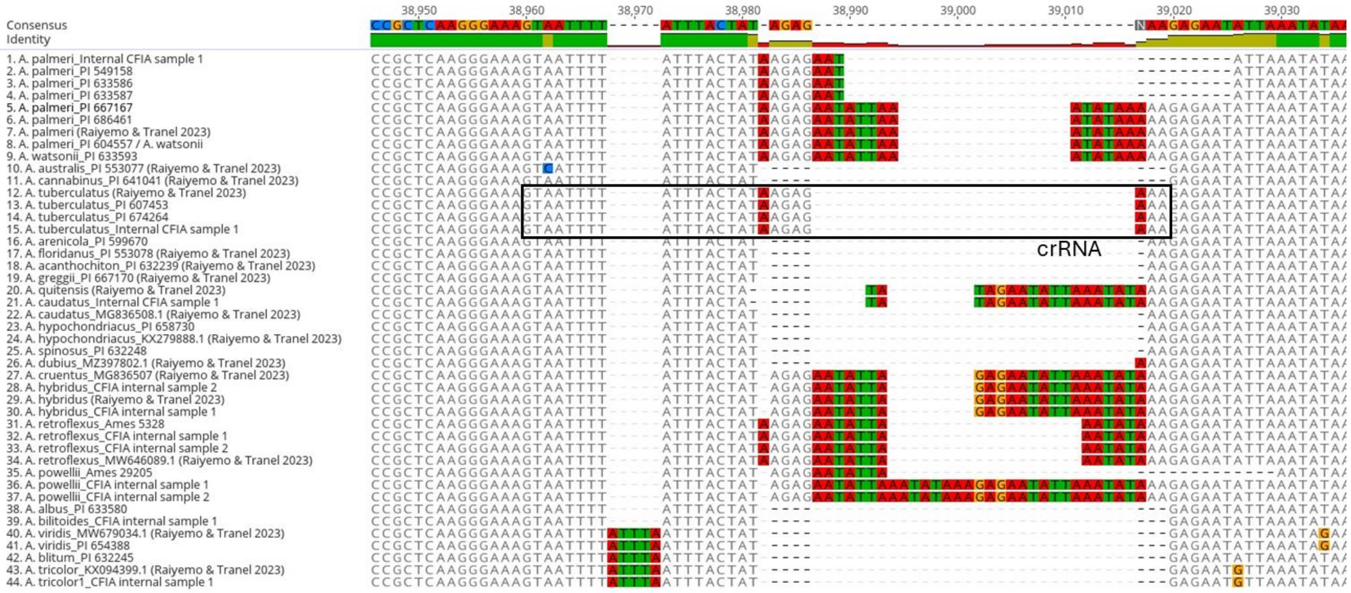

Supplementary Figure 3. *A. palmeri + A. watsonii*-specific CRISPR-LbCas12a fluorescence assay captured every 5 minutes. Sixty-four Amaranthus samples were pre-amplified using recombinase and then individually mixed with LbCas12a, the Apal+Awat_crRNA, and a quenched fluorescent reporter. After 60 minutes of Cas12 reaction only the expected *A. palmeri/A. watsonni* samples showed fluorescence. Number correspond to samples of: *A. albus (1-4), A. arenicola (5-9), A. blitum (10-13), A. blitoides (14-17), A. californicus (18), A. caudatus (19-22), A. hybridus (23-26), A. hypochondriacus (27-31), A. palmeri (32-38), A. powelii (39-43), A. retroflexus (44-47), A. rudis (48), A. spinosus (49-52), A. tricolor (53), A. tuberculatus (54-60), A. viridis (61-63), A. watsonii (64).*

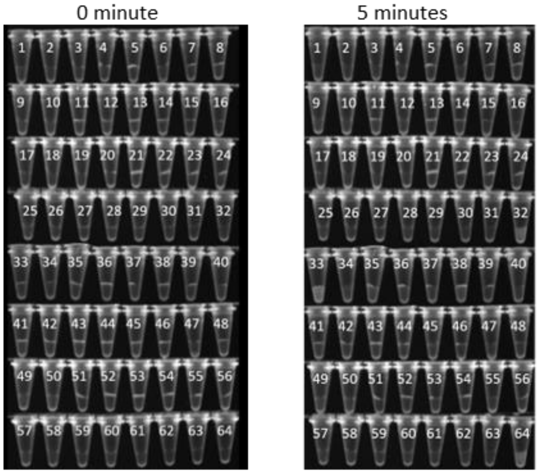

1- MIRL22-Aalb-01
2- MIRL22-Aalb-02
3- MIRL23-Aalb-07
4- MIRL23-Aalb-04
5- MIRL22-Aare-01
6- MIRL23-Aare-02
7- MIRL23-Aare-04
8- MIRL23-Aare-05
9- MIRL23-Aare-03
10- MIRL22-Ablit-04
11- MIRL22-Ablit-03
12- MIRL22-Ablit-06
13- MIRL23-Ablit-07
14- MIRL23-Abli-07
15- MIRL22-Abli-01
16- MIRL23-Abli-05
17- MIRL23-Abli-06
18- MIRL23-Acal-02
19- MIRL23-Acau-09
20- MIRL22-Acau-03
21- MIRL23-Acau-05
22- MIRL23-Acau-06
23- MIRL22-Ahyb-03
24- MIRL23-Ahyb-06
25- MIRL23-Ahyb-05
26- MIRL22-Ahyb-04
27- MIRL22-Ahyp-01
28- MIRL23-Ahyp-17
29- MIRL23-Ahyp-15
30- MIRL23-Ahyp-14
31- MIRL23-Ahyp-11
32- MIRL22-Apal-11
33- MIRL22-Apal-07
34- MIRL22-Apal-06
35- MIRL22-Apal-09
36- MIRL22-Apal-08
37- MIRL22-Apal-10
38- MIRL22-Apal-12
39- MIRL23-Apow-02
40- MIRL22-Apow-03
41- MIRL23-Apow-06
42- MIRL23-Apow-05
43- MIRL22-Apow-04
44- MIRL22-Aret-08
45- MIRL22-Aret-09
46- MIRL23-Aret-12
47- MIRL23-Aret-15
48- MIRL22-Arud-01
49- MIRL22-Aspi-01
50- MIRL23-Aspi-04
51- MIRL22-Aspi-02
52- MIRL23-Aspi-03
53- MIRL22-Atri-05
54- MIRL23-Atub-24
55- MIRL23-Atub-28
56- MIRL22-Atub-17
57- MIRL22-Atub-20
58- MIRL22-Atub-19
59- MIRL22-Atub-18
60- MIRL23-Atub-26
61- MIRL22-Avir-01
62- MIRL23-Avir-02
63- MIRL23-Avir-03
64- MIRL22-Awat-01

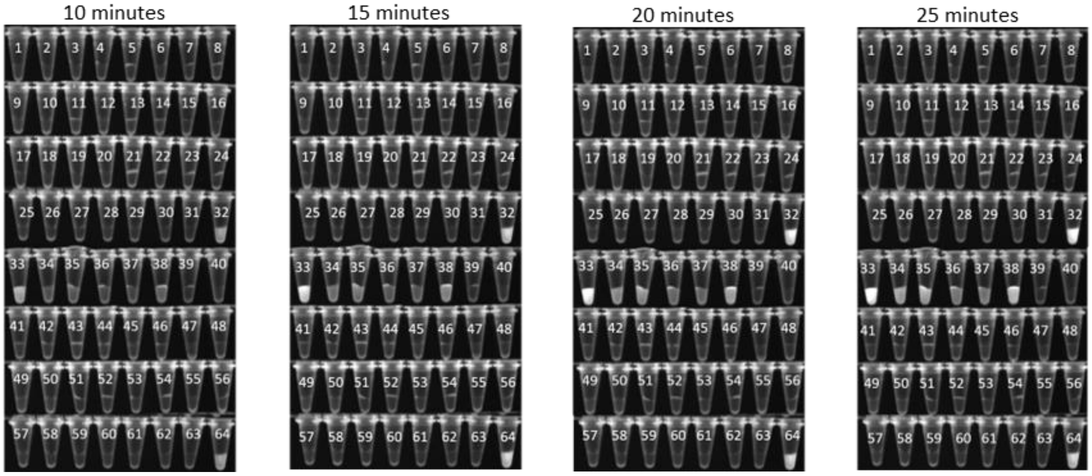

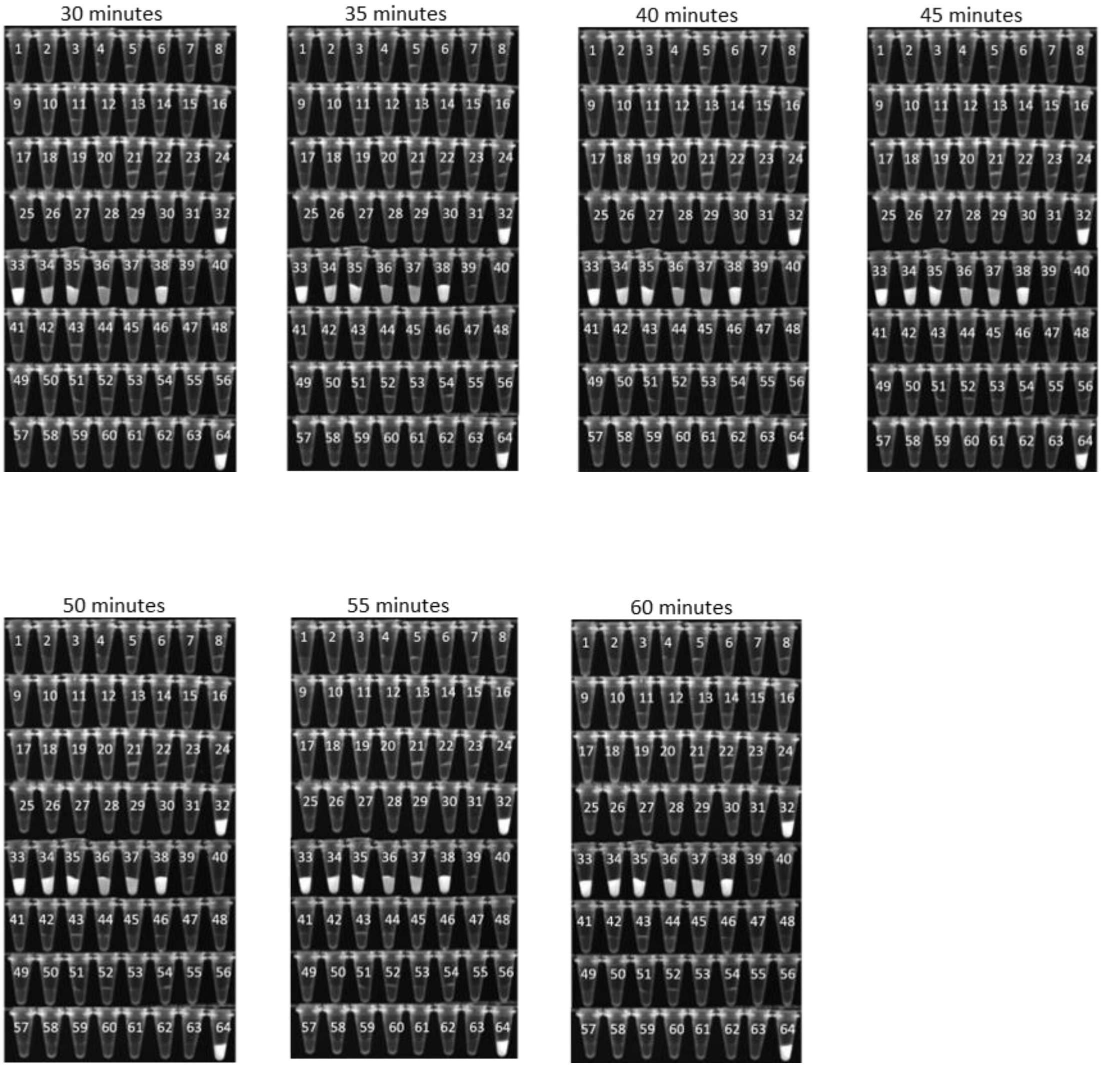

Supplementary Figure 4. Raw fluorescence values of samples corresponding to Supplementary Figure 3.

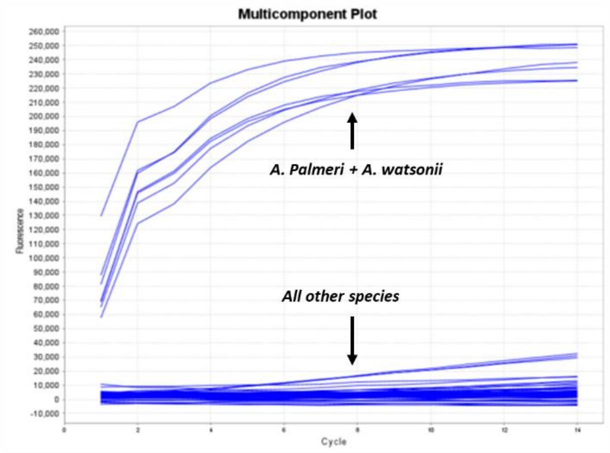

Supplementary Figure 5. *A. palmeri + A. watsonii*-specific CRISPR-LbCas12a fluorescence assay in blind samples provided by the CFIA SSST. Sixty-three Amaranthus samples were preamplified using recombinase and then individually mixed with LbCas12a, an *A. palmeri/A. watsonni*-specific crRNA and a quenched fluorescent reporter. Fluorescence was photographed 60 minutes after the start of the reaction. DNA for sample 1 could not be produced. Samples 1 to 63 correspond to the first 63 samples of Table 2. C1 = positive control *(A. palmeri sample)*, C2 = negative control *(A. tuberculatus)* sample, N – negative control (no DNA).

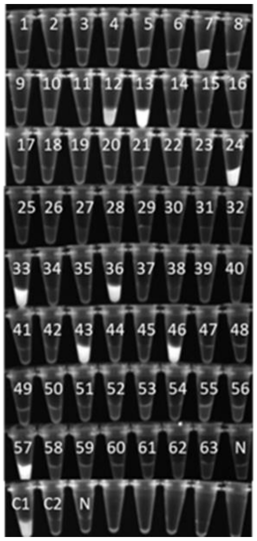

Supplementary Figure 6. A. A preliminary *A. tuberculatus*-specific CRISPR-LbCas12a fluorescence test in our validation set. *Amaranthus* samples were preamplified using recombinase and then individually mixed with LbCas12a, an *A. tuberculatus*-specific crRNA and a quenched fluorescent reporter. **B.** Same preliminary test on blind samples provided by the CFIA SSST. Morphologically identified *A. tuberculatus* samples are in parentheses. Samples 9 and 21 were false negatives. **C.** Positive and false negative samples were sequenced and aligned with previously Illumina-sequenced sections of the target region. The two *A. tuberculatus* false negatives (9 and 21) have a deletion that impairs binding of the crRNA.

A

60 minutes

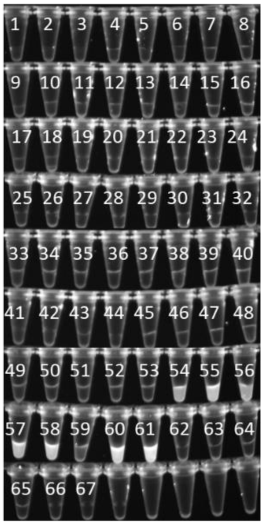

1- MIRL22-Aalb-01
2- MIRL22-Aalb-02
3- MIRL23-Aalb-07
4- MIRL23-Aalb-04
5- MIRL22-Aare-01
6- MIRL23-Aare-02
7- MIRL23-Aare-04
8- MIRL23-Aare-05
9- MIRL23-Aare-03
10- MIRL22-Ablit-04
11- MIRL22-Ablit-03
12- MIRL23-Ablit-06
13- MIRL23-Ablit-07
14- MIRL23-Abli-07
15- MIRL22-Abli-01
16- MIRL23-Abli-05
17- MIRL23-Abli-06
18- MIRL23-Acal-02
19- MIRL23-Acau-09
20- MIRL22-Acau-03
21- MIRL23-Acau-05
22- MIRL23-Acau-06
23- MIRL22-Ahyb-03
24- MIRL23-Ahyb-06
25- MIRL23-Ahyb-05
26- MIRL22-Ahyb-04
27- MIRL22-Ahyp-01
28- MIRL23-Ahyp-17
29- MIRL23-Ahyp-15
30- MIRL23-Ahyp-14
31- MIRL23-Ahyp-11
32- MIRL22-Apal-11
33- MIRL22-Apal-07
34- MIRL22-Apal-06
35- MIRL22-Apal-09
36- MIRL22-Apal-08
37- MIRL22-Apal-10
38- MIRL22-Apal-12
39- MIRL22-Apow-02
40- MIRL22-Apow-03
41- MIRL23-Apow-06
42- MIRL23-Apow-05
43- MIRL22-Apow-04
44- MIRL22-Aret-08
45- MIRL22-Aret-09
46- MIRL23-Aret-12
47- MIRL23-Aret-15
48- MIRL22-Arud-01
49- MIRL22-Aspi-01
50- MIRL23-Aspi-04
51- MIRL22-Aspi-02
52- MIRL23-Aspi-03
53- MIRL22-Atri-05
54- MIRL23-Atub-24
55- MIRL23-Atub-28
56- MIRL22-Atub-17
57- MIRL22-Atub-20
58- MIRL22-Atub-19
59- MIRL22-Avir-01
60- MIRL23-Atub-26
61- MIRL22-Atub-18
62- MIRL23-Avir-02
63- MIRL23-Avir-03
64- MIRL22-Awat-01
65-66-67- Negative

B

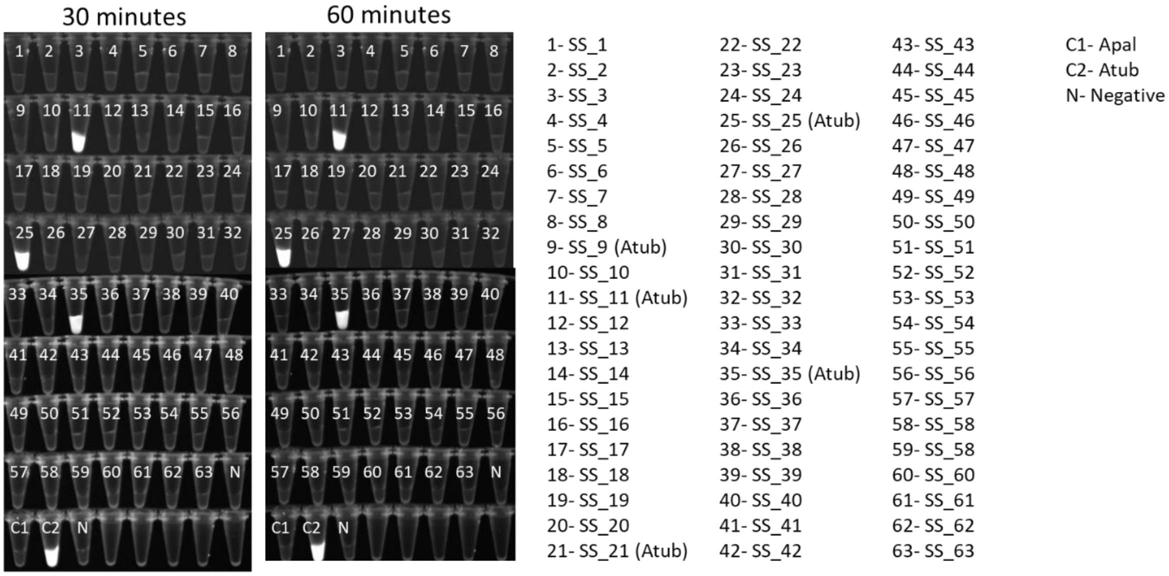
30 minutes
60 minutes
1- SS_1
2- SS_2
3- SS_3
4- SS_4
5- SS_5
6- SS_6
7- SS_7
8- SS_8
9- SS_9 (Atub)
10- SS_10
11- SS_11 (Atub)
12- SS_12
13- SS_13
14- SS_14
15- SS_15
16- SS_16
17- SS_17
18- SS_18
19- SS_19
20- SS_20
21- SS_21 (Atub)
22- SS_22
23- SS_23
24- SS_24
25- SS_25 (Atub)
26- SS_26
27- SS_27
28- SS_28
29- SS_29
30- SS_30
31- SS_31
32- SS_32
33- SS_33
34- SS_34
35- SS_35 (Atub)
36- SS_36
37- SS_37
38- SS_38
39- SS_39
40- SS_40
41- SS_41
42- SS_42
43- SS_43
44- SS_44
45- SS_45
46- SS_46
47- SS_47
48- SS_48
49- SS_49
50- SS_50
51- SS_51
52- SS_52
53- SS_53
54- SS_54
55- SS_55
56- SS_56
57- SS_57
58- SS_58
59- SS_58
60- SS_60
61- SS_61
62- SS_62
63- SS_63
C1- Apal
C2- Atub
N- Negative

C

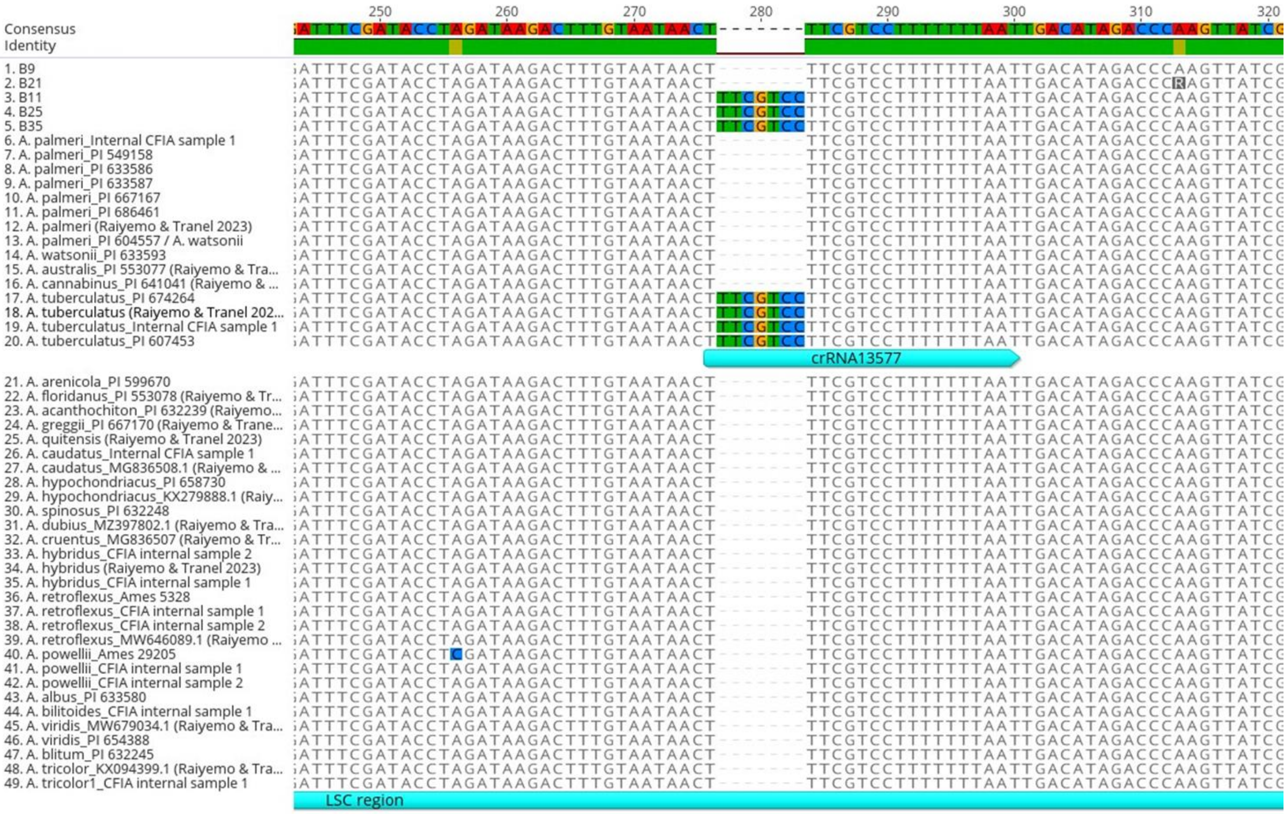
250
260
270
280
290
300
310
320
Consensus
Identity
1. B9
2. B21
3. B11
4. B25
5. B35
6. A. palmeri_Internal CFIA sample 1
7. A. palmeri_PI 549158
8. A. palmeri_PI 633586
9. A. palmeri_PI 633587
10. A. palmeri_PI 667167
11. A. palmeri_PI 686461
12. A. palmeri (Raiyemo & Tranel 2023)
13. A. palmeri_PI 604557 / A. watsonii
14. A. watsonii_PI 633593
15. A. australis_PI 553077 (Raiyemo & Tra...
16. A. cannabinus_PI 641041 (Raiyemo & ...
17. A. tuberculatus_PI 674264
18. A. tuberculatus (Raiyemo & Tranel 202...
19. A. tuberculatus_Internal CFIA sample 1
20. A. tuberculatus_PI 607453
crRNA13577
21. A. arenicola_PI 599670
22. A. floridanus_PI 553078 (Raiyemo & Tr...
23. A. acanthochiton_PI 632239 (Raiyemo...
24. A. greggii_PI 667170 (Raiyemo & Trane...
25. A. quitensis (Raiyemo & Tranel 2023)
26. A. caudatus_Internal CFIA sample 1
27. A. caudatus_MG836508.1 (Raiyemo & ...
28. A. hypochondriacus_PI 658730
29. A. hypochondriacus_KX279888.1 (Raiy...
30. A. spinosus_PI 632248
31. A. dubius_MZ397802.1 (Raiyemo & Tra...
32. A. cruentus_MG836507 (Raiyemo & Tr...
33. A. hybridus_CFIA internal sample 2
34. A. hybridus (Raiyemo & Tranel 2023)
35. A. hybridus_CFIA internal sample 1
36. A. retroflexus_Ames 5328
37. A. retroflexus_CFIA internal sample 1
38. A. retroflexus_CFIA internal sample 2
39. A. retroflexus_MW646089.1 (Raiyemo ...
40. A. powellii_Ames 29205
41. A. powellii_CFIA internal sample 1
42. A. powellii_CFIA internal sample 2
43. A. albus_PI 633580
44. A. blitoides_CFIA internal sample 1
45. A. viridis_MW679034.1 (Raiyemo & Tra...
46. A. viridis_PI 654388
47. A. blitum_PI 632245
48. A. tricolor_KX094399.1 (Raiyemo & Tra...
49. A. tricolor1_CFIA internal sample 1
LSC region

Supplementary Data

Supplementary Data 1. Data and R-script code for statistical analysis of CRISPR-Cas12 fluorescence data 60 minutes after starting the reaction.

#Data was signed-log10-transformed to deal with the negative values of the negative samples (take the absolute value of fluorescence for each sample, perform log 10, and add the negative sign to values that were initially negative). In our case, after averaging the technical replicate fluorescent data, the only negative values were the negative controls. Also, we only used treatments = factors (species) with at least 3 biological replicates (accessions) for statistical testing.

#log10-transformed data (data file: cycle13_60mins_log10_3repsmin.csv)

factor	fluorescence
Aalb	4.04554
Aalb	4.07943
Aalb	4.12174
Aalb	4.14094
Aare	3.84723
Aare	3.63493
Aare	3.82104
Aare	3.63368
Aare	3.38921
Abli	3.66856
Abli	3.62815
Abli	3.95908
Abli	4.15096
Ablit	3.70183
Ablit	3.75443
Ablit	3.75129
Ablit	3.58957
Acau	3.70139
Acau	3.86677
Acau	3.8933
Acau	3.7096
Ahyb	3.95476
Ahyb	3.68637
Ahyb	3.62225
Ahyb	4.17408
Ahyp	3.81049
Ahyp	3.86209
Ahyp	3.95781
Ahyp	3.99156
Ahyp	3.85182
Apal	5.51805
Apal	5.4963
Apal	5.44803
Apal	5.47822
Apal	5.46389
Apal	5.51731
Apal	5.39191
Apow	3.84857

Apow	3.42109
Apow	3.58281
Apow	4.02565
Apow	4.00902
Aret	3.94757
Aret	3.97101
Aret	3.73018
Aret	3.99045
Aspi	3.95249
Aspi	4.40132
Aspi	4.60353
Aspi	4.52955
Atub	3.68812
Atub	3.96567
Atub	3.55689
Atub	3.63097
Atub	3.68371
Atub	3.72619
Atub	3.87342
Avir	3.70222
Avir	3.83024
Avir	3.83504
neg	-3.65385
neg	-3.60192
neg	-3.57288

```
#Set working directory (the code below corresponds to the path to our data)

setwd("PATH:/TO/YOUR_data")


#Load your formatted .csv file

cycle13_60mins_log10_3repsmin <- read.csv("cycle13_60mins_log10_3repsmin.csv", header = TRUE,
stringsAsFactors = FALSE)


#Set your factors (treatments)

cycle13_60mins_log10_3repsmin$factor <- as.factor(cycle13_60mins_log10_3repsmin$factor)


#Test assumptions for statistical tests

#Test normality for residuals with Shapiro-Wilk test

model <- aov(fluorescence ~ factor, data = cycle13_60mins_log10_3repsmin)

res <- residuals(model)

shapiro.test(res)
```

```
#result for Shapiro-Wilk test:

#           Shapiro-Wilk normality test
#data:  res
#W = 0.97671, p-value = 0.2761                    #this means residuals from factors (treatments) are normal


#Test homogeneity of variances with Levene's test

install.packages("car")
library(car)

leveneTest(fluorescence ~ factor, data = cycle13_60mins_log10_3repsmin)

#Result for Levene's test:

#Levene's Test for Homogeneity of Variance (center = median)
#      Df F value  Pr(>F)
#group 13  2.1544 0.02693 *                          #this means variances are not homogeneous
#     49

#Since variances are not homogeneous and the number of replicates (accessions) per factor (species) is different, we need to perform a Welch #ANOVA followed by a post-hoc Games-Howell test.

#Welch ANOVA

library(rstatix)

oneway.test(fluorescence ~ factor, data = cycle13_60mins_log10_3repsmin, var.equal = FALSE)

#result Welch's ANOVA

#One-way analysis of means (not assuming equal variances)
#data:  fluorescence and factor
#F = 5425.5, num df = 13.000, denom df = 16.826, p-value < 2.2e-16                   #significant difference between at least two groups (two species)


#Games-Howell post-hoc comparisons

gh_results <-  cycle13_60mins_log10_3repsmin %>%
    games_howell_test(fluorescence ~ factor)


#start of results:

# A tibble: 91 × 8
#   .y.          group1 group2 estimate conf.low conf.high        p.adj p.adj.signif
# * <chr>        <chr>  <chr>     <dbl>    <dbl>     <dbl>        <dbl> <chr>
# 1 fluorescence Aalb   Aare     -0.432   -0.911   0.0474  0.073        ns
# 2 fluorescence Aalb   Abli     -0.245   -1.13    0.636   0.752        ns
# 3 fluorescence Aalb   Ablit    -0.398   -0.641  -0.154   0.007        **
# 4 fluorescence Aalb   Acau     -0.304   -0.634   0.0259  0.066        ns
```

```
# 5 fluorescence Aalb   Ahyb     -0.238   -1.14    0.668   0.791        ns
# 6 fluorescence Aalb   Ahyp     -0.202   -0.398  -0.00671 0.043        *
# 7 fluorescence Aalb   Apal      1.38     1.25    1.51    0.00000000515 ****
# 8 fluorescence Aalb   Apow     -0.319   -1.03    0.387   0.484        ns
# 9 fluorescence Aalb   Aret     -0.187   -0.590   0.216   0.408        ns
#10 fluorescence Aalb   Aspi      0.275   -0.770   1.32    0.783        ns
# ℹ 81 more rows
# ℹ Use `print(n = ...)` to see more rows
```

```
# write games-Howell results to a .csv file

write.csv(gh_results, "games_howell_results_cycle13_60mins_log10_3respmin.csv", row.names = FALSE)


#Generation of plot to visualize Games-Howell results

#Install packages

install.packages(c("rstatix", "dplyr", "multcompView"))                    #if not installed

library(rstatix)
library(dplyr)
library(multcompView)

summary_stats <- cycle13_60mins_log10_3repsmin %>%
   group_by(factor) %>%
   summarise(mean = mean(fluorescence, na.rm = TRUE),
         sd   = sd(fluorescence, na.rm = TRUE),
         n    = n())

#Build a p-value matrix from Games–Howell results

#Create empty matrix

factors <- levels(cycle13_60mins_log10_3repsmin$factor)

p_mat <- matrix(1, nrow = length(factors), ncol = length(factors),
         dimnames = list(factors, factors))

#Fill in p-values

for (i in 1:nrow(gh_results)) {
   g1 <- as.character(gh_results$group1[i])
   g2 <- as.character(gh_results$group2[i])
   p  <- gh_results$p.adj[i]
   p_mat[g1, g2] <- p
   p_mat[g2, g1] <- p

#Generate grouping letters

letters <- multcompView::multcompLetters(p_mat, threshold = 0.05)$Letters
```

```
#Add letters to summary stats

summary_stats$letters <- letters[summary_stats$factor]

#Plot with ggplot2

ggplot(summary_stats, aes(x = factor, y = mean)) +
    geom_col(fill = "skyblue") +
    geom_errorbar(aes(ymin = mean - sd, ymax = mean + sd), width = 0.2) +
    geom_text(aes(label = letters, y = mean + sd + 0.15), vjust = 0, size = 5) +
    labs(x = "Factor", y = "Mean Fluorescence (log-transformed)") +
    theme_minimal() +
    theme(axis.text.x = element_text(angle = 45, hjust = 1))


#Generation of table to visualize Games-Howell results

#Install packages

install.packages(c("rstatix", "dplyr", "multcompView"))                    #if not installed

library(rstatix)
library(dplyr)
library(multcompView)

summary_stats <- cycle13_60mins_log10_3repsmin %>%
    group_by(factor) %>%
    summarise(mean = mean(fluorescence, na.rm = TRUE),
            sd   = sd(fluorescence, na.rm = TRUE),
            n    = n())

#Build a p-value matrix from Games–Howell results

#Create empty matrix

factors <- levels(cycle13_60mins_log10_3repsmin$factor)

p_mat <- matrix(1, nrow = length(factors), ncol = length(factors),
              dimnames = list(factors, factors))

#Fill in p-values

for (i in 1:nrow(gh_results)) {
    g1 <- as.character(gh_results$group1[i])
    g2 <- as.character(gh_results$group2[i])
    p  <- gh_results$p.adj[i]
    p_mat[g1, g2] <- p
    p_mat[g2, g1] <- p

#Generate compact letter display

letters <- multcompView::multcompLetters(p_mat, threshold = 0.05)$Letters
```

```
#add letters to table

cld_table <- summary_stats %>%
  mutate(group = factor,
        letters = letters[as.character(factor)]) %>%
  select(group, n, mean, sd, letters)

#Export file to .csv

write.csv(cld_table, "CLD_results.csv", row.names = FALSE)
```

Supplementary Data 2. Raw fluorescence values, averaged data and transformed data for samples depicted in Figure 3.

sample ID	Replicate 1	Replicate 2	Replicate 3	factor	fluorescence average - negative samples sign changed	absolute fluorescence log 10 values	fluorescence log 10 transformed (negative values sign changed)
1-MIRL22-Aalb-01	14569.476	10868.056	7878.853	Aalb	11105.461	4.046	4.046
2-MIRL22-Aalb-02	13196.154	11666.025	11158.178	Aalb	12006.786	4.079	4.079
3-MIRL23-Aalb-07	16208.030	13475.679	10022.646	Aalb	13235.451	4.122	4.122
4-MIRL23-Aalb-04	17560.307	14080.108	9861.063	Aalb	13833.826	4.141	4.141
5-MIRL22-Aare-01	6931.764	9367.437	4804.270	Aare	7034.490	3.847	3.847
6-MIRL23-Aare-02	4698.143	6352.838	1892.620	Aare	4314.534	3.635	3.635
7-MIRL23-Aare-04	8157.865	9585.771	2124.525	Aare	6622.721	3.821	3.821
8-MIRL23-Aare-05	4920.599	6281.051	1704.767	Aare	4302.139	3.634	3.634
9-MIRL23-Aare-03	1374.697	5181.960	794.052	Aare	2450.236	3.389	3.389
10-MIRL22-Ablit-04	6010.141	7333.619	1755.497	Ablit	5033.086	3.702	3.702
11-MIRL22-Ablit-03	6421.757	6334.603	4286.662	Ablit	5681.007	3.754	3.754
12-MIRL23-Ablit-06	6338.021	7620.030	2962.209	Ablit	5640.087	3.751	3.751
13-MIRL23-Ablit-07	6263.774	4398.084	997.886	Ablit	3886.581	3.590	3.590
14-MIRL23-Abli-07	6174.518	6550.698	1260.360	Abli	4661.858	3.669	3.669

15-MIRL22-Abli-01	7058.035	6177.510	-492.573	Abli	4247.657	3.628	3.628
16-MIRL23-Abli-05	15411.110	5160.962	6730.071	Abli	9100.714	3.959	3.959
17-MIRL23-Abli-06	17423.773	13063.634	11982.553	Abli	14156.653	4.151	4.151
18-MIRL23-Acal-02	5065.789	6328.258	4821.967	Acal	5405.338	3.733	3.733
19-MIRL23-Acau-09	6887.912	5650.986	2544.829	Acau	5027.909	3.701	3.701
20-MIRL22-Acau-03	10912.610	7431.059	3731.103	Acau	7358.258	3.867	3.867
21-MIRL23-Acau-05	9778.545	8814.390	4872.033	Acau	7821.656	3.893	3.893
22-MIRL23-Acau-06	6774.922	6013.945	2582.658	Acau	5123.842	3.710	3.710
23-MIRL22-Ahyb-03	11560.455	10000.809	5470.697	Ahyb	9010.654	3.955	3.955
24-MIRL23-Ahyb-06	7128.657	5842.129	1600.371	Ahyb	4857.052	3.686	3.686
25-MIRL23-Ahyb-05	4944.823	4907.300	2718.907	Ahyb	4190.343	3.622	3.622
26-MIRL22-Ahyb-04	14436.136	15147.127	15208.887	Ahyb	14930.716	4.174	4.174
27-MIRL22-Ahyp-01	8322.576	7932.083	3136.749	Ahyp	6463.803	3.810	3.810
28-MIRL23-Ahyp-17	8452.334	8385.883	4999.676	Ahyp	7279.298	3.862	3.862
29-MIRL23-Ahyp-15	10199.618	10113.556	6909.436	Ahyp	9074.203	3.958	3.958
30-MIRL23-Ahyp-14	13369.483	11129.309	4923.998	Ahyp	9807.597	3.992	3.992
31-MIRL23-Ahyp-11	9217.751	8066.465	4043.114	Ahyp	7109.110	3.852	3.852
32-MIRL22-Apal-11	392264.031	347237.781	249450.406	Apal	329650.740	5.518	5.518
33-MIRL22-Apal-07	325365.375	364812.438	250457.625	Apal	313545.146	5.496	5.496
34-MIRL22-Apal-06	269148.625	338679.063	233867.563	Apal	280565.083	5.448	5.448
35-MIRL22-Apal-09	362795.813	303023.063	236458.594	Apal	300759.156	5.478	5.478
36-MIRL22-Apal-08	321769.563	326274.563	224958.516	Apal	291000.880	5.464	5.464
37-MIRL22-Apal-10	346734.875	392241.000	248289.375	Apal	329088.417	5.517	5.517
38-MIRL22-Apal-12	171981.688	343288.094	224382.578	Apal	246550.786	5.392	5.392
39-MIRL22-Apow-02	8883.482	8251.233	4033.853	Apow	7056.190	3.849	3.849

40-MIRL22-Apow-03	4415.185	4210.161	-714.664	Apow	2636.894	3.421	3.421
41-MIRL23-Apow-06	2434.329	6636.364	2408.894	Apow	3826.529	3.583	3.583
42-MIRL23-Apow-05	11264.658	13048.521	7512.305	Apow	10608.495	4.026	4.026
43-MIRL22-Apow-04	11243.989	11716.973	7668.557	Apow	10209.840	4.009	4.009
44-MIRL22-Aret-08	13617.463	9128.275	3842.392	Aret	8862.710	3.948	3.948
45-MIRL22-Aret-09	12636.125	9827.915	5598.982	Aret	9354.341	3.971	3.971
46-MIRL23-Aret-12	10927.241	6686.022	-1495.552	Aret	5372.570	3.730	3.730
47-MIRL23-Aret-15	16348.106	9039.304	3959.889	Aret	9782.433	3.990	3.990
48-MIRL22-Arud-01	6577.897	4628.653	2153.193	Arud	4453.248	3.649	3.649
49-MIRL22-Aspi-01	9227.346	10370.544	7293.519	Aspi	8963.803	3.952	3.952
50-MIRL23-Aspi-04	5588.080	40108.547	29889.820	Aspi	25195.482	4.401	4.401
51-MIRL22-Aspi-02	50192.266	42009.367	28206.277	Aspi	40135.970	4.604	4.604
52-MIRL23-Aspi-03	40728.770	33495.848	27322.756	Aspi	33849.124	4.530	4.530
53-MIRL22-Atri-05	23400.336	14217.767	15110.863	Atri	17576.322	4.245	4.245
54-MIRL23-Atub-24	4640.898	6274.719	3714.364	Atub	4876.660	3.688	3.688
55-MIRL23-Atub-28	11706.653	10283.247	5730.156	Atub	9240.019	3.966	3.966
56-MIRL22-Atub-17	5561.910	3494.470	1758.337	Atub	3604.906	3.557	3.557
57-MIRL22-Atub-20	4149.837	4169.542	4506.536	Atub	4275.305	3.631	3.631
58-MIRL22-Atub-19	6283.623	5034.115	3164.426	Atub	4827.388	3.684	3.684
59-MIRL22-Atub-18	7300.522	4999.351	3670.242	Atub	5323.372	3.726	3.726
60-MIRL23-Atub-26	10205.427	8102.081	4107.699	Atub	7471.735	3.873	3.873
61-MIRL22-Avir-01	6539.453	5796.393	2776.760	Avir	5037.535	3.702	3.702
62-MIRL23-Avir-02	8125.162	8483.494	3685.051	Avir	6764.569	3.830	3.830
63-MIRL23-Avir-03	8790.872	7111.945	4616.533	Avir	6839.784	3.835	3.835
64-MIRL22-Awat-01	365406.625	314048.563	250547.484	Awat	310000.891	5.491	5.491
65-neg 1	-4621.363	-4787.177	-4111.343	neg	4506.628	3.654	-3.654

66-neg 2	-3364.031	-5021.552	-3610.434	neg	3998.672	3.602	-3.602
67-neg 3	-2995.014	-4033.743	-4191.514	neg	3740.090	3.573	-3.573
